# Advancements in Carbon‐Based Piezoelectric Materials: Mechanism, Classification, and Applications in Energy Science

**DOI:** 10.1002/adma.202419970

**Published:** 2025-04-25

**Authors:** Mude Zhu, Qingyou Liu, Wai‐Yeung Wong, Linli Xu

**Affiliations:** ^1^ Department of Applied Biology and Chemical Technology and Research Institute for Smart Energy The Hong Kong Polytechnic University Hung Hom Kowloon Hong Kong 999077 P. R. China; ^2^ School of Materials and Environmental Engineering Shenzhen Polytechnic University Shenzhen 518055 P. R. China

**Keywords:** biomedical innovations, carbon‐based piezoelectric materials, energy harvesting, piezoelectric catalysis, piezoelectric coefficients, sensors

## Abstract

The piezoelectric phenomenon has garnered considerable interest due to its distinctive physical properties associated with the materials involved. Piezoelectric materials, which are inherently non‐centrosymmetric, can generate an internal electric field under mechanical stress, enhancing carrier separation and transfer due to electric dipole moments. While inorganic piezoelectric materials are often investigated for their high piezoelectric coefficients, they come with potential drawbacks such as toxicity and high production cost, which hinder their practical applications. Consequently, carbon‐based piezoelectric materials have emerged as an alternative to inorganic materials, boasting advantages such as a large specific surface area, high conductivity, flexibility, and eco‐friendliness. Research into the applications of carbon‐based piezoelectric materials spans environmental remediation, energy conversion, and biomedical treatments, indicating a promising future. This review marks the first comprehensive attempt to discuss and summarize the various types of carbon‐based piezoelectric materials. It delves into the underlying mechanisms by which piezoelectricity influences catalysis, biomedical applications, nanogenerators, and sensors. Additionally, various potential techniques are presented to enhance the piezoelectric performance. The design principles of representative fabrication strategies for carbon‐based piezoelectric materials are analyzed, emphasizing their current limitations and potential improvements for future development. It is believed that recent advances in carbon‐based piezoelectric materials will make a significant impact.

## Graphical abstract

Carbon‐based piezoelectric materials are systematically categorized based on their structural and functional properties. The mechanisms of stress‐induced charge transfer are elucidated, and their applications are explored across three key domains: piezoelectric catalysis for energy conversion and environmental remediation, piezoelectric biomedical treatments for tissue regeneration and antitumor therapies, and piezoelectric generators and sensors for energy harvesting and advanced sensing technologies.

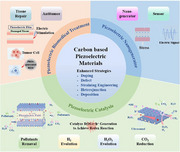

## 1 Introduction

Piezoelectric materials, characterized by their non‐centrosymmetric structures, can generate a polarization effect through the induction of polarized charges in opposing domains upon the application of an external mechanical stress. This phenomenon leads to the generation of an internal electric field, enabling the use of these materials in transistors, light‐emitting diodes (LEDs), catalysts, and solar cells.^[^ ^1^, ^2^, ^3^, ^4^ ^]^ The piezoelectric effect, originating at the molecular level, manifests macroscopically as the generation of internal positive and negative charges when certain dielectric materials are subjected to compressive or tensile stress along a specific crystallographic direction. The density of these induced charges is directly proportional to the magnitude of the applied force.^[^ ^5^, ^6^, ^7^ ^]^ Among the 32 crystallographic point groups, 20 lack a center of symmetry, which is a prerequisite for piezoelectric behavior. Most common crystalline materials, however, exhibit symmetric structures. When an external force is applied to a piezoelectric material, it induces a relative shift of the positive and negative ions within the unit cell, leading to charge misalignment and the creation of an electric dipole moment. Consequently, this induces macroscopic polarization within the crystal, giving rise to a piezoelectric potential,^[^ ^8^ ^]^ where positive and negative charges are driven to opposite ends of the material. Under tensile or compressive stress, a piezoelectric material generates opposing charges at each end, with the direction of the polarized electric fields oriented oppositely. In the absence of an external force, piezoelectric materials do not exhibit a net polarized electric field because the atomic positions within the unit cell result in charge neutralization.^[^ ^9^ ^]^ For example, in unstressed ZnO, zinc and oxygen ions are tetrahedrally coordinated, with the centers of positive and negative charge aligned, resulting in no net polarization.^[^ ^10^ ^]^ Upon application of stress, zinc and oxygen atoms are displaced from their equilibrium positions, causing the positive and negative charges to separate and migrate in opposite directions within the crystal, leading to dipolar polarization and the creation of an intrinsic electric field.

In recent years, various materials have been explored for piezoelectric catalysis and sensing applications due to their unique properties. These include wurtzite materials such as ZnO and CdS,^[^ ^11^, ^12^, ^13^, ^14^ ^]^ perovskite materials like BaTiO_3_, PbTiO_3_, CuCo_2_S_4_, and KNbO_3_,^[^ ^15^, ^16^, ^17^, ^18^ ^]^ bismuth‐based materials including BiFeO_3_ and BiO*X* (where *X* = Cl, Br, I),^[^ ^19^, ^20^, ^21^ ^]^ and transition metal dichalcogenides such as MoS_2_ and WS_2_.^[^ ^22^, ^23^, ^24^, ^25^ ^]^ The piezoelectric effect, when induced by external forces, can drive the transfer of intrinsic charge carriers to the surface, generating an electric current and voltage suitable for nanogenerators. Additionally, redox reactions with absorbed molecules can occur on the surface, facilitating catalytic transformations. Therefore, these piezoelectric materials can harness renewable vibrational energy, converting it into electrical and chemical energy, offering environmentally friendly approaches for chemical synthesis, biomedical treatments, pollutant removal, water splitting, and CO_2_ reduction.^[^ ^26^, ^27^, ^28^, ^29^ ^]^ For instance, BaTiO_3_ has been employed to facilitate arylation and borylation reactions via ball milling,^[^ ^30^ ^]^ tumor treatments,^[^ ^31^ ^]^ and has achieved 100% dye degradation along with a H_2_ production rate of 0.899 mmol g^−1^ h^−1^ under ultrasonic conditions.^[^ ^32^, ^33^ ^]^ However, many reported piezoelectric materials are limited to inorganic compounds produced at an experimental scale, constrained by high raw material costs and time‐consuming manufacturing processes.

Carbon‐based materials, owing to their abundance, cost‐effectiveness, and environmental compatibility, are well‐suited for large‐scale applications. These materials encompass a range of structures, including graphitic carbon nitride (g‐C_3_N_4_), organic polymers, metal–organic frameworks (MOFs), covalent organic frameworks (COFs), graphdiyne (GDY), graphene, and carbon nanotubes (CNTs). They play a crucial role in piezocatalysis due to their high specific surface area, flexibility, excellent electrical conductivity, significant mechanical strength, and robust chemical stability, positioning them as compelling candidates for piezoelectric‐driven reactions.^[^ ^34^, ^35^, ^36^ ^]^ The high specific surface area of these carbon‐based materials provides abundant active sites for redox reactions, while their superior conductivity facilitates efficient charge transfer kinetics. Furthermore, their mechanical strength is crucial for withstanding stresses associated with piezocatalysis, particularly the intense forces generated by ultrasonically induced cavitation, which could otherwise compromise the structural integrity of materials lacking sufficient mechanical resilience. For instance, 2D conjugated microporous polymers, such as Zn‐Salen‐TEPT, exhibit a high specific surface area of 420 m^2^ g^−1^ and a high electrical conductivity of 1.362 µS cm^−1^ while demonstrating an exceptional piezoelectric activity for H_2_ evolution at 3260 µmol g^−1^ h^−1^ under ultrasound. Importantly, their structure remains stable after six cycling tests.^[^ ^37^ ^]^ Carbon‐based piezoelectric materials offer notable advantages over traditional inorganic materials in terms of biocompatibility and reduced toxicity, especially when appropriately functionalized. Careful consideration of their physicochemical properties, such as surface functional groups and defect density, is essential to ensure safety in biomedical applications. In contrast, traditional inorganic materials, such as lead‐zirconate‐titanate (PZT) and BaTiO_3_, have established uses but face challenges related to toxicity, primarily concerning lead content.^[^ ^31^ ^]^ External forces can induce structural deformation in these materials, leading to an uneven distribution of electron cloud density, thereby inducing piezoelectricity. Although pristine 2D graphene, with its nearly zero bandgap and symmetric structure, typically does not exhibit piezoelectric properties,^[^ ^38^ ^]^ recent studies have shown that graphene can exhibit a band piezoelectric effect when subjected to a biaxial strain. This alters graphene's electronic structure and leads to a potential gradient within cavities, demonstrating a piezoelectric coefficient of 37 nC N^−1^, surpassing that of many traditional crystalline piezoelectric materials. Additionally, surface piezoelectric effects, induced by uneven charge distribution under strain, have been experimentally confirmed in multi‐walled carbon nanotubes.^[^ ^39^, ^40^ ^]^ Similar to inorganic piezoelectric materials, the piezoelectric properties of carbon materials can also be enhanced by doping with heteroatoms or introducing significant defects and morphological engineering, as these modifications promote improved charge carrier separation and transfer.^[^ ^41^, ^42^ ^]^ For example, bulk g‐C_3_N_4_, tubular g‐C_3_N_4_ (TCN), and porous tubular g‐C_3_N_4_ (PTCN) can be easily prepared via hydrothermal methods or high‐temperature calcination. Among these forms, PTCN exhibits a superior piezoelectric performance, with 100% removal of rhodamine B (Rh B) achieved in just 3 min under piezoelectric photocatalysis. This enhanced performance is attributed to its porous tubular structure that absorbs more vibrational energy, thereby increasing the piezoelectric potential, which accelerates carrier mobility as they react with molecules to form radicals that decompose pollutants.^[^ ^43^ ^]^ Additionally, many piezoelectric organic polymers are biocompatible and biodegradable, thus they can be applied in biomedical contexts. For instance, self‐aligned piezoelectric *γ*‐glycine/polyvinyl alcohol (PVA) films can achieve 40% wound healing under ultrasound while being self‐degradable without side effects in murine models.^[^ ^44^ ^]^ Consequently, incorporating carbon‐based materials into piezoelectric applications opens up new avenues for developing efficient, sustainable, and cost‐effective catalytic systems.

**Figure 1:**
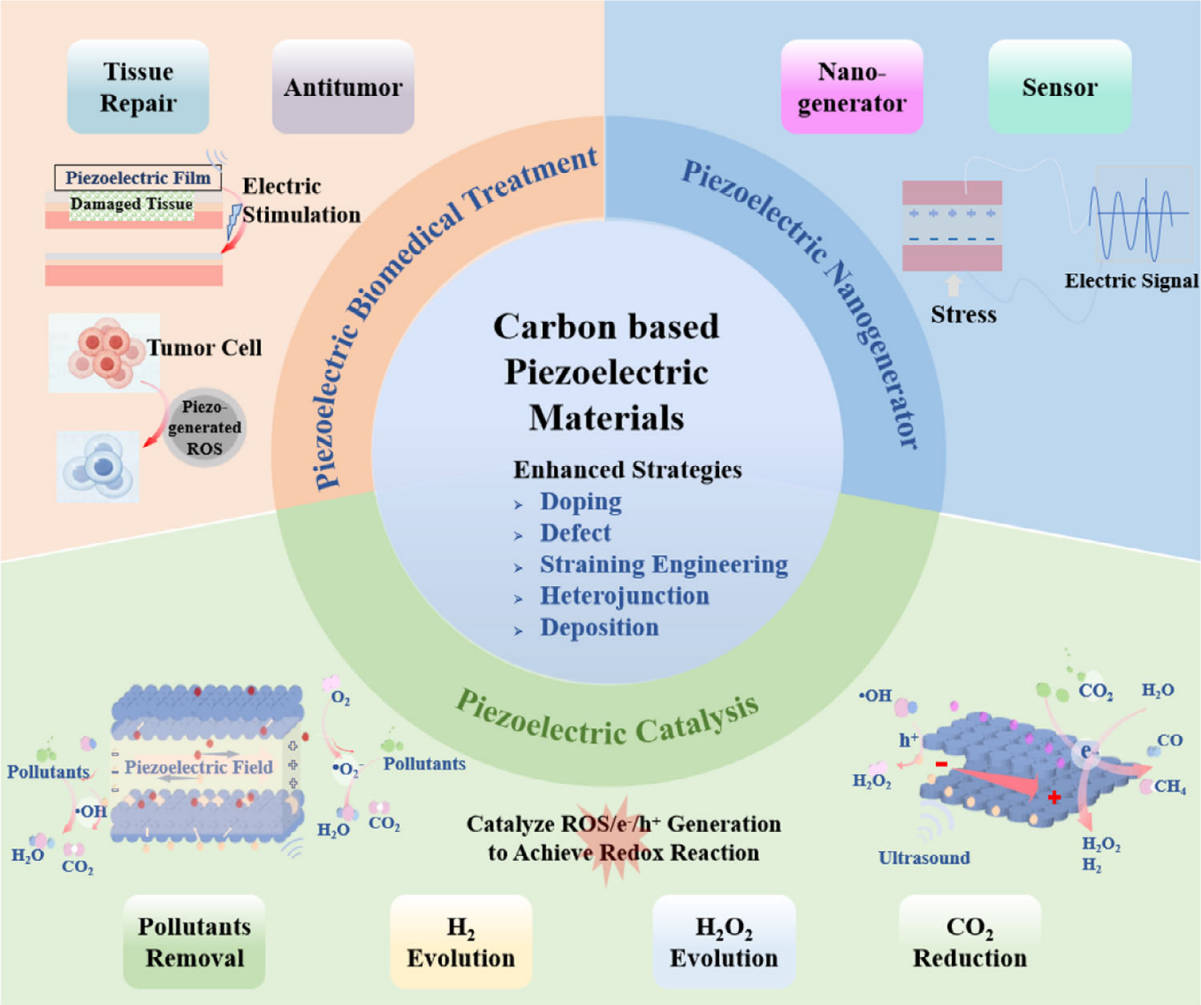
The piezoelectric mechanism, classification, and piezoelectric applications of carbon‐based piezoelectric materials.

To date, some reviews have focused on applications and mechanisms related to piezoelectric catalysis, biomedical applications, and nanogenerators. Chen et al. discussed various inorganic and organic materials for biomedical applications.^[^ ^29^ ^]^ Yang et al. summarized advancements in piezoelectric photocatalysis for water splitting,^[^ ^45^ ^]^ while Rajaboina et al. focused on porous organic materials for nanogenerators.^[^ ^46^ ^]^ Herein, we present a comprehensive review of recent advancements in carbon‐based piezoelectric materials, along with their mechanisms and applications. The initial section delves into the development of carbon‐based materials for piezoelectricity, highlighting various types of these materials along with their distinctive structures, and summarizing mechanisms related to piezoelectric catalysis as well as biomedical treatments and nanogenerators, while the corresponding energy band theory concepts, such as charge screening effects, alongside charge flow dynamics are clarified. The subsequent section delves into the practical applications of these piezoelectric materials exploring their underlying mechanisms and diverse use. Piezoelectric catalysis plays a crucial role in energy conversion and environmental remediation by harnessing mechanical energy to drive free radical reaction, which are pivotal in initiating chemical transformations and degradation processes. Additionally, piezoelectric biomedical treatments leverage electric stimulation to enhance tissue regeneration and antitumor therapies, capitalizing on the ability for these materials to generate electrical signals in response to mechanical stress. Furthermore, piezoelectric generators and sensors are utilized for energy harvesting and advanced sensing technologies, converting environmental vibrations into usable electrical energy through stress‐induced electric signals. These applications are visually represented in **Figure** **1**, which provides an overview of the multifaceted roles of piezoelectric materials in energy science and biomedical engineering. Finally, we critically evaluate recent advancements concerning potential applications alongside current limitations faced by these materials, while highlighting strategies aimed at improvement. Additionally, we outline emerging opportunities within this field while providing insights for future research endeavors.

## 2 Carbon‐Based Piezoelectric Materials

Carbon‐based materials, characterized by their high specific surface areas, tunable physicochemical properties, and environmentally benign preparation methods, are emerging as promising candidates in the field of piezoelectricity.

### 2.1 Graphitic Carbon Nitride (g‐C_3_N_4_)

g‐C_3_N_4_ is a polymeric semiconductor featuring a tri‐s‐triazine structure with a graphitic π‐conjugated framework. g‐C_3_N_4_ exhibits high stability at room temperature and under high pressure, along with inherent semiconducting properties.^[^ ^34^ ^]^ It offers several advantages, including good chemical stability, tunable electronic structure, n‐type semiconductivity, and earth‐abundance constituents.^[^ ^47^ ^]^ From a microstructural perspective, g‐C_3_N_4_ possesses a graphite‐like layered architecture. The aromatic heterocyclic units within the layers, combined with van der Waals forces between the layers, confer chemical and thermal stability to g‐C_3_N_4_.^[^ ^48^ ^]^ Additionally, the graphite‐like layered structure endows g‐C_3_N_4_ with a high specific surface area. Theoretical calculations suggest that an ideal monolayer of the g‐C_3_N_4_ sample can achieve a specific surface area of up to 2500 m^2^ g^−1^.^[^ ^49^ ^]^

**Figure 2:**
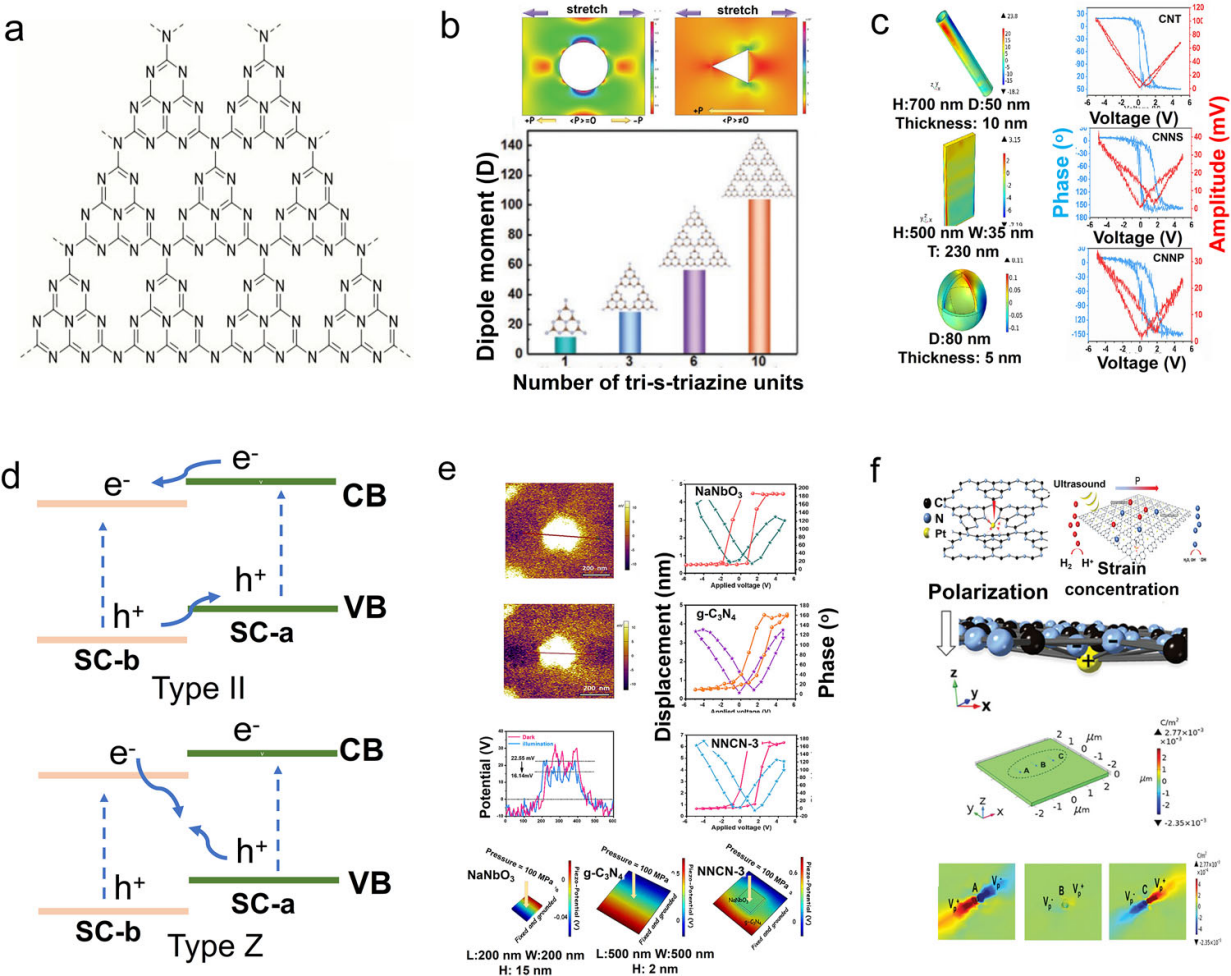
a) The geometric structure of g‐C_3_N_4_. b) Finite element method (FEM) simulations illustrating the stress distribution around smooth planes, circular pores, and triangular pores in tri‐*s*‐triazine sheets of g‐C_3_N_4_, along with the piezoelectric response under varying stress conditions and the dipole moment of the g‐C_3_N_4_ monolayer as a function of the number of tri‐*s*‐triazine units along the *a*‐axis. Reproduced with permission.^[^ ^48^ ^]^ Copyright 2021, Wiley‐VCH. c) Piezoelectric behavior associated with different g‐C_3_N_4_ morphologies. Reproduced with permission.^[^ ^54^ ^]^ Copyright 2023, Elsevier. d) Schematic representation of a type II heterojunction and a Z‐scheme heterojunction, illustrating the energy band alignment and charge transfer pathways in each configuration. e) Piezoelectric polarization facilitating carrier transfer and enhancing the piezoelectric response in the C_3_N_4_/NaNbO_3_ heterojunction. Reproduced with permission.^[^ ^58^ ^]^ Copyright 2023, Elsevier. f) Polarization changes induced by single Pt atom deposition on g‐C_3_N_4_, Reproduced with permission.^[^ ^67^ ^]^ Copyright 2023, Wiley‐VCH.

It is generally accepted that g‐C_3_N_4_ comprises two primary structural units: the triazine ring (C_3_N_3_), associated with the *R*3m space group, and the heptazine ring (C_6_N_7_) associated with the *P*6m2 space group.^[^ ^50^ ^]^ However, density functional theory (DFT) calculations indicate that the binding energy of the heptazine unit is lower than that of the triazine unit, suggesting that the heptazine unit exhibits a thermodynamically more stable structure.^[^ ^51^ ^]^ Consequently, g‐C_3_N_4_ predominantly consists of heptazine structural units, which are inherently non‐centrosymmetric (**Figure** **2**a).

As an organic semiconductor, the electronic structure and band distribution of g‐C_3_N_4_ are of particular interest. In 2D g‐C_3_N_4_, carbon (C) and nitrogen (N) atoms are evenly distributed in a plane, forming a hexagonal network through σ bonds. A highly delocalized π‐conjugated framework is established by lone pairs of electrons on N atoms in the *p*_z_ orbital due to *sp* ^2^ hybridization.^[^ ^52^ ^]^ The formation of this π‐conjugated system is believed to be responsible for the catalytic activity of g‐C_3_N_4_. The lone pairs of N atoms primarily contribute to the formation of the highest occupied molecular orbital, while the lone pairs of C atoms constitute the lowest unoccupied molecular orbital, corresponding to valence band (VB) and conduction band (CB) in semiconductors, respectively. Therefore, g‐C_3_N_4_ has been developed as a photocatalyst for pollutant degradation, CO_2_ reduction, and H_2_ evolution due to its abundant active sites and suitable electronic band structure. Notably, its unique nanometer‐scale triangular holes are non‐centrosymmetric. This piezoelectricity was initially verified through combined theoretical and experimental research conducted by Matthew Zelisko et al., with the use of a powerful‐technique, piezoresponse force microcopy (PFM), confirming that g‐C_3_N_4_ is indeed a piezoelectric material.^[^ ^53^ ^]^ The flexoelectric effect and non‐centrosymmetric holes are major contributors to its piezoelectricity. In Figure 2b, when a regular non‐piezoelectric sheet with circular holes is subjected to uniform stretching, local polarization can be induced due to flexoelectricity.^[^ ^48^ ^]^ However, the entire sheet does not exhibit piezoelectric behavior because positive and negative charge centers coincide within circular holes. In contrast, for a non‐centrosymmetric hole, net average polarization is generated under an external force, leading to piezoelectricity. The in‐plane piezoelectric coefficient can reach 2.18 C m^−1^, exceeding that of many inorganic piezoelectric materials like hexagonal boron nitride (*h*‐BN) (1.38 C m^−1^).

#### 2.1.1 Enhancing the Piezoelectricity of g‐C_3_N_4_

Enhancing the piezoelectric properties of g‐C_3_N_4_ is a significant area of research due to its potential to expand the material's applicability in advanced technological fields, such as catalysis, energy harvesting, and sensing. Enhancing the material's response to mechanical stimuli can generate stronger current signals and promote the formation of reactive radicals, thereby improving performance in applications such as pollutant removal. Several strategies are employed to enhance the piezoelectricity of g‐C_3_N_4_, including morphology engineering, heterojunction formation, doping, and deposition.

##### Morphology Engineering

Morphology engineering involves employing various synthetic strategies, such as adding surfactants and adjusting the proportions of fluxing agents, to control the growth of specific crystal facets, thereby exposing desired surface terminations. Different semiconductor materials and preparation methods lead to distinct morphological characteristics, enabling the catalysts to exhibit unique geometries and electronic structures that result in varying piezoelectric performances. As shown in Figure 2b, thermal oxidation etching of bulk g‐C_3_N_4_ can produce ultrathin g‐C_3_N_4_ nanosheets (UT‐g‐C_3_N_4_), which demonstrate enhanced linear piezoelectricity. The piezoelectric coefficient of UT‐g‐C_3_N_4_ was calculated to be ≈524 pm V^−1^, which is 132 pm V^−1^ greater than that of bulk g‐C_3_N_4_, as further verified by finite element method (FEM) analysis. Additionally, DFT calculations clarified that increasing the tri‐s‐triazine units along the *a*‐axis contributes to enhanced piezoelectric polarization. Wu et al. applied hydrothermal and high temperature solid‐phase methods to prepare bulk g‐C_3_N_4_, tubular g‐C_3_N_4_ (TCN), and porous tubular g‐C_3_N_4_ (PTCN). Among these, PTCN exhibited the largest specific surface area of 53.42 m^2^ g^−1^, enabling it to readily absorb molecules and capture external vibrational energy. FEM further clarified that PTCN possesses the strongest piezopotential under 100 MPa stress.^[^ ^43^ ^]^ Similarly, Wang et al. utilized controlled vapor deposition methods to synthesize hollow nanotubes (CNNTs), nanosheets (CNNSs), and hollow nanospheres (CNNPs) from g‐C_3_N_4_. PFM verified that CNNTs exhibited superior piezoelectric performance, with a piezoelectric coefficient *d*_33_ of 34.99 pm V^−1^, while FEM calculations indicated a piezoelectric potential of 42 V, better than that of CNNSs (10.34 V) and CNNPs (0.25 V) (see Figure 2c).^[^ ^54^ ^]^

##### Constructing Heterojunctions

The principle behind constructing heterostructures involves combining two or more semiconductor materials based on favorable energy band alignment to create an energy band offset between them. This promotes efficient electron and hole transfer between conduction and valence bands.^[^ ^55^ ^]^ A potential difference forms between the semiconductors, facilitating charge carrier separation. Therefore, constructing heterojunctions can effectively enhance the separation and migration efficiency of photogenerated carriers, improving the photocatalytic activity. Common heterojunction configurations include type II and Z‐scheme heterojunctions (Figure 2d).^[^ ^56^, ^57^ ^]^ In type II heterojunctions, both the valence band and conduction band positions of semiconductor **a (SC‐a)** are higher than those of semiconductor **b** (**SC‐b**). The difference in chemical potential between **SC‐a** and **SC‐b** leads to an energy band bending at their interface, inducing the formation of a built‐in electric field that drives photogenerated electrons and holes to migrate in opposite directions, resulting in spatial charge separation. Therefore, forming type II heterostructures is an effective method to improve charge separation efficiency. In Z‐type heterojunction, during the excitation process, electrons in the CB of **SC‐b** recombine with holes in the VB of **SC‐a**. The remaining electrons and holes then reside in the CB of **SC‐a** and VB of **SC‐b**, respectively. This configuration enhances charge carrier separation efficiency and results in high redox capability. Xu. et al. constructed a type II heterojunction using g‐C_3_N_4_/g‐C_3_N_4‐_ *_x_* S*_x_*. In this system, g‐C_3_N_4−_ *_x_* S*_x_* has a more negative potential which allows holes to transfer into its CB while electrons migrate from g‐C_3_N_4_ into the CB of g‐C_3_N_4−_ *_x_* S*_x_*, thereby enhancing the redox activity of the composite.^[^ ^47^ ^]^ Under ultrasonic conditions, excited electrons and holes could be accelerated in their transfer due to the induced piezoelectric fields and interface electric fields, leading to improved overall piezoelectric activity. Additionally, g‐C_3_N_4_ often forms composites with other piezoelectric semiconductors to further enhance the piezoelectric performance, as exemplified by NaNbO_3_/g‐C_3_N_4_ (Figure 2e).^[^ ^58^ ^]^ According to Kelvin probe force microscopy (KPFM) and PFM results, both materials exhibit enhanced piezoelectric polarization effects when combined. The overall piezoelectric displacement significantly increases from ≈4.5 nm for NaNbO_3_ and 3.7 nm for g‐C_3_N_4_ to 6.5 nm for NaNbO_3_/g‐C_3_N_4_. FEM further demonstrates the enhanced piezoelectric potential of NaNbO_3_/g‐C_3_N_4_ at 0.67 V compared with NaNbO_3_ at 0.58 V and g‐C_3_N_4_ at 0.45 V. Other composites, such as g‐C_3_N_4_/Pt‐PVDF, Bi_2_MoO_6_/C_3_N_4_ and Cr/Nb modified Bi_4_Ti_3_O_12_/g‐C_3_N_4_, have also been explored in this context.^[^ ^59^, ^60^, ^61^ ^]^

##### Deposition Techniques

The deposition of metal or non‐metal species onto a primary substrate is a widely adopted strategy in photocatalysis to enhance the catalytic performance. This technique not only improves light absorption but also accelerates the separation and transfer of charge carriers.^[^ ^62^, ^63^ ^]^ By leveraging the benefits of deposition, many piezoelectric materials are modified with specific substances, such as noble metals like Au and Ag, to augment their piezoelectric performance.^[^ ^64^, ^65^ ^]^ The underlying mechanism is analogous to that observed in photocatalysis. The deposition of a secondary material can enhance the generation of a piezoelectric potential, thereby increasing the internal driving force that facilitates the separation and transfer of electron–hole pairs. Furthermore, the deposited material can act as a trap or barrier for electrons or holes, preventing their recombination and thus enhancing the efficiency of piezoelectric catalysis. Additionally, the deposited material can serve as active sites, improving the interface contact between external molecules and the composite material. Consequently, electrons or holes can be more effectively transferred to the reaction sites, thereby enhancing the piezoelectric catalytic reaction. For instance, Yan et al. designed carbon quantum dots (CQDs) deposited onto g‐C_3_N_4_, which exhibits excellent piezoelectric catalytic degradation activity for Rh B.^[^ ^66^ ^]^ First, the deposition of CQDs increases the surface area, providing numerous active sites that enhance interactions between the material and Rh B molecules. Second, CQDs can function as electron capture sites that promote electron accumulation, leading to an inhomogeneous surface potential distribution that further enhances piezoelectricity. Finally, the introduction of CQDs can act as electron transfer mediators for piezoelectrically generated electrons, facilitating their transfer and thereby contributing to enhanced piezoelectric catalytic degradation. Moreover, Pt single atom‐deposited g‐C_3_N_4_ demonstrates superior piezocatalytic H_2_ evolution activity because Pt single atoms coordinate with nearby C and N atoms, significantly disrupting charge symmetry within g‐C_3_N_4_. This enhanced asymmetry improves polarization ability and accelerates carrier transfer and separation. This phenomenon has been investigated through theoretical calculation (see Figure 2f).^[^ ^67^ ^]^

##### Doping

Doping is an effective strategy to influence the catalytic properties of a material by introducing heteroatoms.^[^ ^41^ ^]^ The substitution sites for different types of ions vary. Specifically, doping with metal ions creates impurity energy levels within the electronic band structure, reducing the energy required for electrons to undergo transition to the CB and thereby improving the separation efficiency and migration performance of electron–hole pairs. Introducing non‐metals into the semiconductor allows hybridization with their energy bands, which can reduce the bandgap of the semiconductor. Consequently, overall piezoelectric performance may be enhanced. For example, doping with ammonium molten salts (NH_4_ *X*, where *X* = F, Cl, Br) leads to the formation of nitrogen vacancy (NV) defect states that not only reduce the required bandgap energy but also create multiple electron channels to accelerate the separation and transfer of electrons and holes.^[^ ^68^ ^]^ Similarly, Se‐doped g‐C_3_N_4_ possesses additional defect energy levels that act as electron mediators to inhibit carrier recombination. Thus, catalytic activity can be significantly enhanced.^[^ ^41^ ^]^

### 2.2 Piezoelectric Polymers

Polymer piezoelectric materials have been extensively studied due to their inherent advantages, such as flexibility, lightweight properties, excellent processing capabilities, and the ability to create large‐area and curved surfaces. They are also effective in converting weak mechanical energy into electrical energy.^[^ ^69^ ^]^ Compared to inorganic materials, polymers offer benefits such as toughness, non‐toxicity, and biocompatibility, making them suitable for applications that require environmental friendliness and high bending and twisting capabilities in fields such as environmental remediation, energy conversion, and biomedical devices.^[^ ^70^, ^71^ ^]^

#### 2.2.1 PVDF

**Figure 3:**
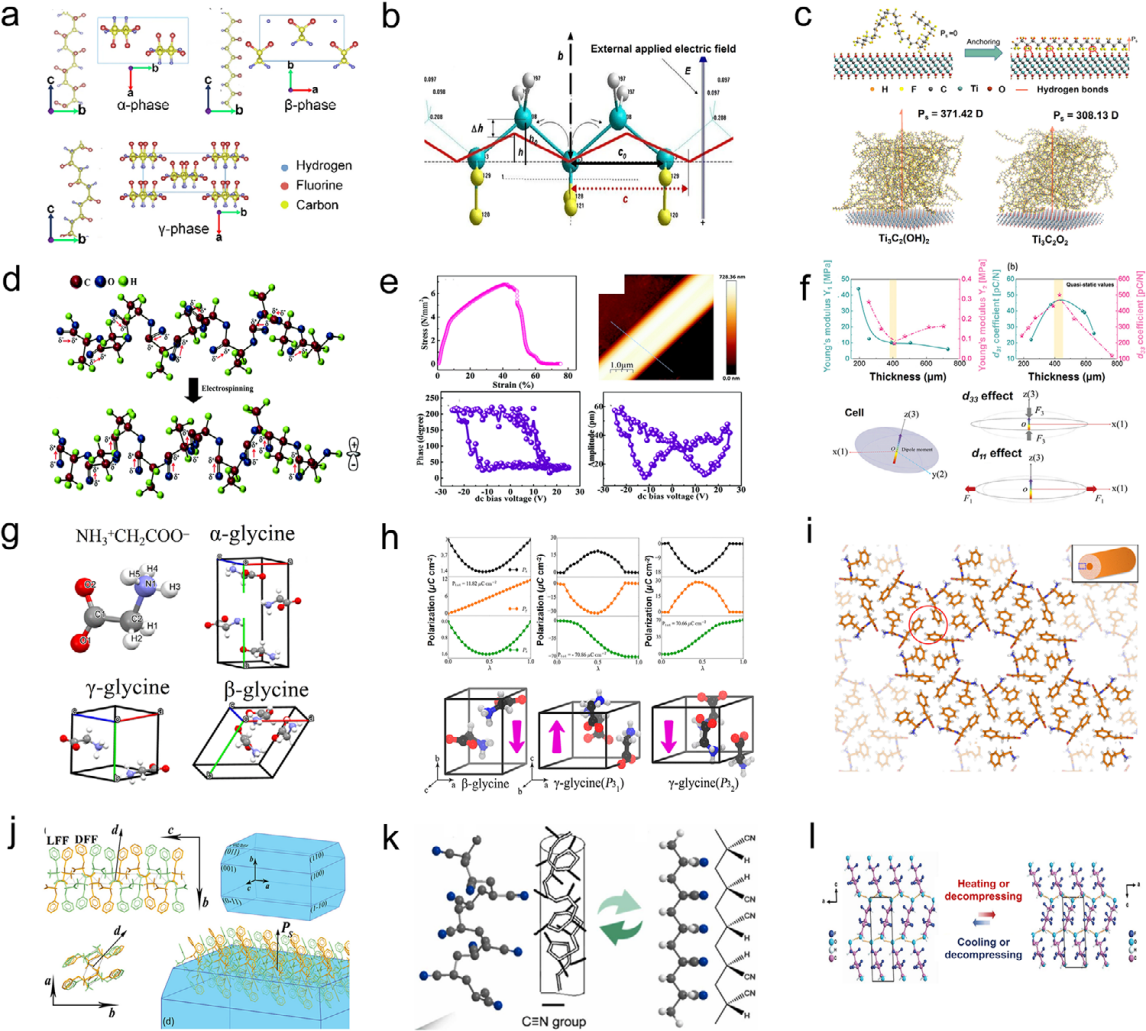
a) Crystal structures and chain packing arrangements of the three primary polymorphs of PVDF: *α‐*, *β‐*, *γ*‐phases. Reproduced with permission.^[^ ^75^ ^]^ Copyright 2024, Springer‐Verlag. b) Deformation of a segment of the PVDF chain skeleton under an externally applied electric field. Reproduced with permission.^[^ ^76^ ^]^ Copyright 2013, Springer‐Verlag. c) Enhanced piezoelectric polarization resulting from the incorporation of combination of Ti_3_C_2_T*x* MXene nanosheets into a PVDF matrix. Reproduced with permission.^[^ ^80^ ^]^ Copyright 2022, The Authors, published by Springer Nature. d) Changes in the molecular structure of the PLLA chain and the orientation of C═O dipoles induced by electrospinning. Reproduced with permission.^[^ ^95^ ^]^ Copyright 1998, The Japan Society of Applied Physics. e) Piezoelectric response characterization of PLLA. Reproduced with permission.^[^ ^96^ ^]^ Copyright 2017, The Royal Society of Chemistry. f) Influence of film thickness on the mechanical properties and piezoelectric response of PLLA ferroelectrets. Reproduced with permission.^[^ ^97^ ^]^ Copyright 2023, The Authors, published by Wiley‐VCH. g) Molecular dipole orientations in *α‐*, *β‐*, *γ*‐glycine crystalline structures. Reproduced with permission.^[^ ^99^ ^]^ Copyright 2020, American Chemical Society. h) MPT pathway illustrating variations in the polarization of *β*‐glycine, *γ*‐glycine (*P*3_1_), and *γ*‐glycine (*P*3_2_) during structural transitions. Reproduced with permission.^[^ ^100^ ^]^ Copyright 2019 American Chemical Society. i) Simulated crystal structure of FF peptide nanotubes. Reproduced with permission.^[^ ^102^ ^]^ Copyright 2013, American Chemical Society. j) Orientation of spontaneous polarization in layered biomolecular crystals of LDFF. Reproduced with permission.^[^ ^107^ ^]^ Copyright 2021, Wiley‐VCH. k) Phase transition of PAN chains. Reproduced with permission.^[^ ^110^ ^]^ Copyright 2023, Elsevier. l) Phase transition of HEFP under thermal or stress field. Reproduced with permission.^[^ ^113^ ^]^ Copyright 2024, The Authors, published by Science.

Piezoelectric polymers can be categorized into amorphous, crystalline, and semi‐crystalline structures. Among these, polyvinylidene fluoride (PVDF) is classified as a semi‐crystalline piezoelectric polymer composed of long‐chain units that exhibit a net dipole moment due to the electronegative fluorine atoms bonding with positive hydrogen atoms.^[^ ^72^ ^]^ PVDF contains piezoelectric active components and can be synthesized into various forms, including nanostructures, wires, fibers, tubes, and more. Compared to other commercial polymer materials, PVDF demonstrates superior mechanical strength, variability, chemical resistance, reinforcement capabilities, high heat resistance, biocompatibility, and thermal stability. It is widely used in energy harvesters, batteries, sensors, and biomedical applications.^[^ ^73^ ^]^ PVDF has five polymorphs: *α‐*, *β‐*, *γ‐*, *δ‐*, and *ε*‐phases. These phases are associated with monoclinic (*P*2_1_/*c*), orthorhombic (*Cm*2*m*), and orthorhombic (*P*2_1_ *cn*) structures, as shown in **Figure** **3**a. Among them, the *α‐*, *β‐*, *γ*‐phases exhibit piezoelectric properties. The *β*‐phase (TTTT), characterized by the highest dipolar moment per unit cell, exhibits greater piezoelectricity than the *α*‐(TGTG’) and *γ*‐(T3GT3G’) phases.^[^ ^74^, ^75^ ^]^ In molecular modeling and first‐principle calculations (Figure 3b), it was observed that the length of the PVDF chain increases when an external electric field is applied along the dipole moment. The piezoelectric coefficient *d*_33_ ranges from −16.6 to −19.2 pC N^−1^, demonstrating the excellent piezoelectric properties of *β*‐PVDF.^[^ ^76^ ^]^ The *β*‐phase is particularly important due to its high electrical property and superior piezoelectricity and ferroelectricity. Therefore, optimizing piezoelectricity and ferroelectricity to achieve a high *β*‐phase fraction in PVDF is a priority. Since *α*‐, *β*‐, and *γ*‐phases can interconvert, applying pressure, heat, or electric field can facilitate the formation of PVDF with optimal piezoelectric properties. Additionally, factors such as stretch ratio, temperature, crystallinity, and poling conditions can significantly influence the piezoelectric performance.^[^ ^77^ ^]^ It has also been reported that the piezoelectric coefficient of PVDF can reach 23 pC N^−1^,^[^ ^78^ ^]^ which is superior to that of some inorganic materials such as aluminum nitride, zinc oxide, and quartz.

PVDF exhibits excellent characteristics as a piezoelectric material, including a low thermal conductivity and a low dielectric constant, which contribute to very high electric field constants.^[^ ^79^ ^]^ Despite these advantages, its inherent limitations, such as a relatively low piezoelectric charge constant (*d*_33_) and a low dielectric constant, still restrict its application in piezoelectric catalysis. Various approaches have been employed to increase the *β*‐phase concentration to enhance the piezoelectricity of PVDF, including metal loading, copolymerization with PVDF derivatives, and metal oxide doping. Su et al. reported that anchoring Ti_3_C_2_T*_x_* MXene could improve the *β*‐phase fraction (Figure 3c).^[^ ^80^ ^]^ The molecular chain of fluoropolymers is initially curved, resulting in low piezoelectricity due to a small *β*‐phase content. However, OH surface termination on Ti_3_C_2_T*_x_* facilitates hydrogen bonding with fluorine bonds on the fluoropolymer matrix, leading to aligned fluoropolymer units and enhanced spontaneous polarization in polymer‐ceramic composites. Similarly, CdS can enhance the *β*‐phase fraction through coordination between Cd^2+^ ions and F^−^ ions. Concurrently, this heterojunction can generate a built‐in electric field that accelerates electron transfer under the combined effects of the piezoelectric field and built‐in electric field.^[^ ^81^ ^]^ Furthermore, copolymers of PVDF, including poly(vinylidene fluoride hexafluoro propylene) (PVDF‐HFP), poly(vinylidene fluoride trifluoroethylene) (P(VDF‐TrFE)), poly(vinylidene cyanide‐vinyl acetate) (P[VDCN‐VAC]), and poly(vinylidene fluoride tetrafluoroethylene) (P[VDF‐TeFE]), have been developed and demonstrated excellent piezoelectric performance.^[^ ^82^, ^83^, ^84^, ^85^ ^]^

#### 2.2.2 PTFE

Polytetrafluoroethylene (PTFE) is a polymer formed by the polymerization of tetrafluoroethylene, resulting in a non‐centrosymmetric structure. It has been reported that PTFE exhibits excellent chemical stability, high corrosion resistance, low dielectric constant, and high thermal stability. Additionally, the piezoelectric coefficient *d*_33_ can reach 220 pC N^−1^ under an applied pressure of 6.24 kPa.^[^ ^86^ ^]^ Wang et al. investigated the piezoelectric properties of PTFE under various treatments. Initially, the piezoelectric response is quite low. However, it increases significantly after processes such as stretching, compaction, and electric treatment.^[^ ^87^ ^]^ This enhancement occurs because PTFE is a nonpolar polymer electret material that generates polarization in response to an external stimuli. Compared with PVDF, PTFE exhibits superior piezoelectric properties. Furthermore, PTFE can generate various reactive oxygen species (ROS), including H_2_O_2_, ^1^O_2_, ^•^O_2_^−^, and ^•^OH. Due to its favorable piezoelectric characteristics, PTFE has been utilized for dye degradation at a rate of 0.246 min^−1^ and for tooth whitening under ultrasound. This effectiveness is attributed to the piezoelectric‐generated ROS decomposing dye molecules and bacteria.^[^ ^88^, ^89^ ^]^ Composites of Fe^0^ and PTFE (Fe@PTFE) have been developed to enhance ^•^OH generation under ultrasound.^[^ ^90^ ^]^ The presence of Fe^2+^ facilitates the reduction of H_2_O_2_ into ^•^OH, which possesses stronger oxidizing properties, thereby improving the piezoelectric degradation capability. Enhanced piezoelectricity can also be achieved by constructing PTFE@TiO_2_ composites, where fluorine (F) atoms transfer electrons to oxygen (O) atoms. This interaction results in a built‐in electric field at the interface between PTFE and the TiO_2_ surface, leading to band bending and significant local dipole enhancement in PTFE containing F vacancies.^[^ ^91^ ^]^

#### 2.2.3 Biodegradable Piezoelectric Polymers

Most inorganic and some organic materials lack inherent biocompatibility, which can lead to biotoxicity, rendering these types of piezoelectrics unsuitable for certain medical devices. Recently, the emergence of biodegradable piezoelectric materials composed of small organic molecules presents an opportunity to address the e‐waste problem while enhancing the biocompatibility of devices. Furthermore, biodegradable materials can be utilized to develop implantable medical devices designed to function within the body for a specific duration.^[^ ^70^, ^92^ ^]^ This category of materials shares similar physical characteristics with PVDF, including flexibility, ease of synthesis, and favorable piezoelectric properties.

Polylactic acid (PLLA) is among the most extensively studied biodegradable piezoelectric polymers. This transparent and highly flexible plant‐derived polymer is considered environmentally benign. PLLA can be synthesized through l‐lactide ring‐opening polymerization.^[^ ^93^ ^]^ PLLA exhibits three crystallization phases: *α*, *β*, and *γ*. The *α*‐crystalline phase of PLLA is thermodynamically stable, with C═O dipoles randomly oriented along the polymer backbone, resulting in no net piezoelectricity.^[^ ^94^ ^]^ PLLA has a complex hierarchical structure that induces both crystalline and amorphous regions, allowing for modulation of crystallinity. Consequently, the piezoelectric properties of PLLA films can be designed and enhanced by increasing crystallinity and molecular orientation. To induce and enhance piezoelectricity, polymer chains can be thermally stretched or subjected to electrospinning processes, converting the *α*‐crystalline form into the *β*‐crystalline form. This transformation results in a shift from randomly oriented molecular chains to C═O dipoles aligned along the stretching direction (Figure 3d).^[^ ^95^ ^]^ The piezoelectric properties were verified using PFM, which revealed characteristic 180° phase‐reversing phase–voltage curves and butterfly‐like amplitude–voltage curves (Figure 3e). The *d*_33_ coefficient was determined to be 3 ± 1 pm V^−1^, while PLLA has a *d*_14_ coefficient of 10 pC N^−1^.^[^ ^96^ ^]^ The primary piezoelectric polarization in PLLA ferroelectrets was investigated through Young's modulus versus thickness measurements (Figure 3f). The change in axial strain‐induced *d*_33_ effect is analogous to the axial stretch‐induced *d*_31_ effect, with maximum values ≈500 pC N^−1^ for *d*_33_ and 48 pC N^−1^ for *d*_31_.^[^ ^97^ ^]^ Thus, *d*_33_ plays a dominant role in determining piezoelectricity.

Glycine is an amino acid with an achiral structure that has three crystalline polymorphs: *α*‐glycine, *β*‐glycine, and *γ*‐glycine (Figure 3g).^[^ ^98^, ^99^ ^]^ The antiparallel molecular dipoles in the *α*‐glycine crystal structure, belonging to space group *P*2_1_/n, results in no net polarization and, thus, no piezoelectricity for this polymorph.^[^ ^98^ ^]^ In contrast, *β*‐glycine and *γ*‐glycine structures have space groups of *P*2_1_ and *P*3_1_ or *P*3_2_, respectively, exhibiting favorable piezoelectric properties. The *d*_33_ constant for the *𝛾*‐glycine has been measured at 10 pm V^−1^ using PFM, while the shear piezoelectricity *d*_16_ of *β*‐glycine reaches 178 pm V^−1^.^[^ ^98^, ^99^ ^]^ DFT calculations and PFM confirm that both 𝛾‐glycine and *β*‐glycine possess ferroelectric properties that classify them as piezoelectric materials.^[^ ^100^, ^101^ ^]^ In Figure 3h, along the transition path, the glycine molecule rotates 180° around an axis perpendicular to the *c*‐axis. The polarization of the two *γ*‐glycine phases is approximately five times greater than that of *β*‐glycine. In *β*‐glycine, the dipoles of different molecules are in different directions, whereas in *γ*‐glycine, the moments are helically aligned, and the net components are arranged along the *c*‐axis. The non‐coincidence of the negative charge centers within their unit cell structure leads to a permanent built‐in electric field that generates an electric dipole moment not equal to zero, resulting in spontaneous polarity in the crystals. While *β*‐glycine exhibits better ferroelectricity than 𝛾‐glycine. 𝛾‐glycine possesses helical dipoles along its axis that allow for stronger and more controllable polarization compared to *β*‐glycine's dipoles oriented in different directions.^[^ ^99^ ^]^ These unique properties have attracted significant attention for applications in energy conversion and electronic information.

Diphenylalanine (FF) peptide nanotubes (PNTs) have been successfully synthesized as a piezoelectric material composed of amino acids (Figure 3i).^[^ ^102^ ^]^ Atomistic replica exchange molecular dynamics simulations have been employed to investigate structural changes in FF peptides under an external electric field.^[^ ^103^ ^]^ It has been reported that piezoelectric shear constant *d*_15_ for FF PNTs exceeds 60 pm V^−1^.^[^ ^104^ ^]^ However, achieving macroscopic piezoelectricity in FF PNTs is challenging because polarization arises from orientation and size rather than uniform unidirectional growth. To enhance macroscopic piezoelectricity, epitaxially uniform FF microrods with hexagonally arranged nanochannels have been synthesized. These exhibit a *d*_33_ value of 9.9 pm V^−1^.^[^ ^105^ ^]^ Furthermore, polarization in FF microrods can be enhanced by applying an external voltage during self‐assembly, increasing the *d*_33_ constant to 17.9 pm V^−1^.^[^ ^106^ ^]^ Zelenovski et al. synthesized a layered biomolecular crystal of FF through co‐assembly of l, l‐ and l, l‐enantiomers of FF monomers, referred to as LDFF. Each LDFF asymmetric unit contains two LFF and two DFF monomers connected by hydrogen bond interactions. The polarization ability of LDFF is lower than that of FF monomers due to differing polarization directions that partially offset the overall polarization electric field (Figure 3j).^[^ ^107^ ^]^ Nonetheless, its out‐of‐plane piezoelectricity *d*_33_ measured by PFM can still reach 20 pm V^−1^.

Polyacrylonitrile (PAN), characterized by a large dipole moment due to the cyano group on its side chain, is a promising piezoelectric polymer material that has garnered significant attention.^[^ ^108^, ^109^ ^]^ The PAN chain structure is depicted in Figure 3k. It is initially irregular, which limits its piezoelectric properties. However, through thermal polling, mechanical stretching, or electrospinning processes, dipoles can be efficiently oriented. This orientation leads to a planar zigzag formation of the PAN chain that enhances its piezoelectricity.^[^ ^110^ ^]^ Applied external forces such as strain can regulate the distance or direction between atoms or molecules, resulting in changes to the electric dipole moment. Consequently, both polarization intensity and direction vary. The *d*_33_ coefficient for PAN ranges from 2 to 10 pC N^−1^ depending on temperature changes. PAN‐based piezoelectric elastomers can even achieve values up to 40 pC N^−1^, illustrating excellent tunability.^[^ ^111^, ^112^ ^]^

A novel ferroelectric molecular crystal, HOCH_2_(CF_2_)_3_CH_2_OH (HFDP), was synthesized. This compound is interconnected through O─H···O hydrogen bonding with adjacent molecules (Figure 3l).^[^ ^113^ ^]^ The asymmetric C─F bond imparts spontaneous polarization ability to the molecule. The measured piezoelectric coefficient *d*_33_ can reach up to 138 pC N^−1^. Additionally, HFDP‐PVA exhibits excellent biocompatibility and biodegradability. Under an impulse force of 40 N, this film can generate an output voltage of ≈18 V and functions as a self‐powered transient energy harvesting device for therapeutics applications in mice.

### 2.3 MOFs/COFs

**Figure 4:**
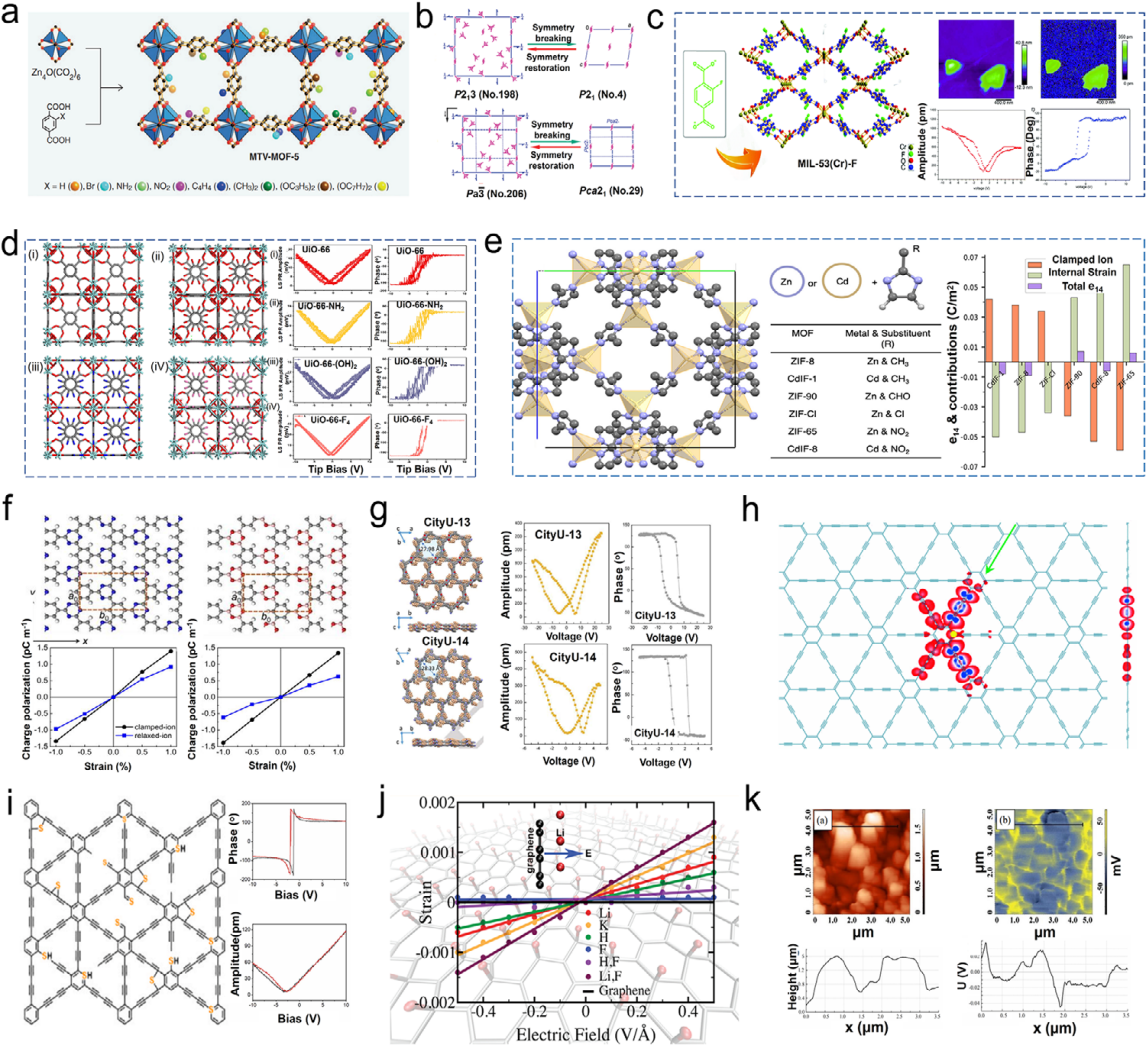
a) The structure of MTV‐MOF‐5. Reproduced from permission.^[^ ^114^ ^]^ Copyright 2013, Science. b) Transformations of the space group of [CH_3_NH_3_][M(H_2_O)_6_](SO_4_)_2_·6H_2_O from the paraelectric phase to the ferroelectric phase. Reproduced with permission.^[^ ^119^ ^]^ Copyright 2012, American Chemical Society. c) The fabrication and piezoelectric properties of MIL‐53(Cr)‐F. Reproduced with permission.^[^ ^125^ ^]^ Copyright 2021, The Royal Society of Chemistry. d) The structure and piezoelectric response of UiO‐66, UiO‐66‐NH_2_, UiO‐66‐(OH), and UiO‐66‐F_4_. Reproduced with permission.^[^ ^156^ ^]^ Copyright 2023, Elsevier. e) Representation of the unit cell of ZIFs and their corresponding piezoelectric constants. Reproduced with permission.^[^ ^127^ ^]^ Copyright 2022, The Authors, published by American Chemical Society. f) Optimized structures of C_9_H_3_N_3_ and C_6_H_3_B_3_O_3_ monolayers and the relationships between charge polarization and the uniaxial strain applied. Reproduced with permission.^[^ ^131^ ^]^ Copyright 2017, AIP Publisher. g) The piezoelectricity of City‐U COFs as measured by PFM. Reproduced with permission.^[^ ^132^ ^]^ Copyright 2024, The Authors, published by Wiley‐VCH. h) The distribution of electron density of GDY. Reproduced with permission.^[^ ^135^ ^]^ Copyright 2011, American Physical Society. i) The piezoelectricity of S‐doped GDY. Reproduced with permission.^[^ ^142^ ^]^ Copyright 2022, Elsevier. j) An external electric field applied perpendicular to the graphene sheet induces an equiaxial strain in the plane of the sheet. Reproduced with permission.^[^ ^148^ ^]^ Copyright 2011, American Chemical Society. k) The surface potential of strained CNTs.^[^ ^154^ ^]^ Copyright 2018, IOP Publisher.

Metal‐organic frameworks (MOFs), also known as porous coordination polymers, constitute a class of porous materials constructed by linking inorganic and organic units through strong chemical bonds to form 1D, 2D, and 3D structures, as exemplified by MTV‐MOF‐5 shown in **Figure** **4**a.^[^ ^114^ ^]^ MOFs possess several distinctive properties: 1) Tunable design: MOFs can be rationally designed through the coordination of metal ions or metal clusters with organic linkers. The structure of the resulting MOFs is determined by the geometry of the metal clusters and the shape of the organic linkers. For example, many MOFs can be formed by combining M_2_(CO_2_)_4_ (where M = Cu, Zn, Fe, Mo, Cr, Co, or Ru) with various tritopic organic linkers, such as benzene‐1,3,5‐tricarboxylate.^[^ ^115^ ^]^ 2) Microporosity and high surface area: Porous MOFs exhibit microporous characteristics, with pore sizes typically ranging from a few angstroms to several nanometers. This is commonly achieved by controlling the length of bi‐ or multi‐dentate rigid organic linkers. MOFs exhibit ultra‐high porosity, with free volumes reaching up to 90% and internal surface areas exceeding 10 000 m^2^ g^−1^ (Langmuir surface area).^[^ ^116^ ^]^ Consequently, there are a vast number of active sites within MOFs, allowing for the modulation of immobilized functional metal sites. 3) Thermal and chemical stability: MOFs demonstrate high thermal and chemical stability due to their strong chemical bonds, including C─C, C─H, C─O, and metal─O bonds.^[^ ^117^ ^]^ Some MOFs can tolerate high temperatures ranging from 250 to 500 °C, Zeolitic imidazolate framework‐8 (ZIF‐8) and certain MOFs, such as Zr_6_O_4_(OH)_4_(BDC)_6_, exhibit outstanding resistance to strong acids and bases.^[^ ^118^ ^]^ It has been reported that many MOFs exhibit piezoelectric properties due to their polar space group transformations (Figure 4b). Examples include [(NH_4_)[Zn(HCOO)_3_], [(CH_3_)_2_NH_2_][M(HCOO)_3_] (where M = divalent Mn, Fe, Co, Ni, Zn), [Mn_3_(HCOO)_6_]_3_·C_2_H_5_OH], [Cu(HCOO)_2_(H_2_O)_2_]_3_^•^2H_2_O, and [Ag(NH_3_CH_2_COO)(NO_3_)]. However, their ferroelectric properties typically manifest at high or low temperatures, which is not favorable for catalytic applications.^[^ ^119^ ^]^ Fortunately, MOFs such as MIL‐53(Cr)‐F, UIO‐66, MIL‐100(Fe), ZIF‐8, and UiO‐66‐NH_2_ have been developed and successfully applied in piezoelectric catalysis.^[^ ^120^, ^121^, ^122^, ^123^, ^124^ ^]^ MIL‐53(Cr)‐F consists of a polar linker containing fluorine‐substituted terephthalic acid. PFM measures a maximum piezoelectric amplitude of 1051 pm (Figure 4c).^[^ ^125^ ^]^ Zhao et al. synthesized a series of piezoelectric MOFs, including UiO‐66, UiO‐66‐NH_2_, UiO‐66‐(OH)_2_, and UiO‐66‐F_4_, by reacting glacial acetic acid (HAC) and zirconium tetrachloride (ZrCl_4_) with 1,4‐dicarboxybenzene (BDC), 2,5‐dihydroxyterephthalic acid (BDC‐(OH)_2_), 2‐aminoterephthalic acid (BDC‐NH_2_), and tetrafluoroterephthalic acid (BDC‐F_4_). Among these, UiO‐66‐F_4_ exhibited superior piezoelectric properties, as measured by PFM (Figure 4d).^[^ ^126^ ^]^ The high porosity and large specific surface area of MOFs can enhance their piezoelectricity by increasing the amount of charge that can be stored. The tunability of their properties also allows for optimization of their piezoelectric response. The piezoelectric properties of a MOF can be enhanced by selecting metal ions and organic ligands that increase its polarizability.^[^ ^119^ ^]^ Zeolitic imidazolate frameworks (ZIFs) with various metal nodes and linker substituents have been constructed, and their polarization abilities were calculated using systematic DFT calculations (Figure 4e).^[^ ^127^ ^]^ The piezoelectric coefficient *d*_14_ ranges from −38 to −46 pC N^−1^ for CdIF‐1, −9 to −14 pC N^−1^ for ZIF‐8, <±8 pC N^−1^ for ZIF‐90, ZIF‐Cl and ZIF‐65, and 2 to 50 pC N^−1^ for CdIF‐8.

Covalent organic frameworks (COFs) consist of crystalline, porous organic polymers in which organic building blocks are connected through strong covalent bonds to form well‐defined 2D or 3D structures with long‐range order.^[^ ^128^ ^]^ The strong covalent bonds result in low mass density, high thermal stability, and permanent porosity. 2D COFs facilitate charge carrier transport in the stacking direction. Meanwhile, 3D COFs containing *sp* ^3^ carbon or silane atoms possess high specific surface area, numerous active sites, and low density.^[^ ^129^ ^]^ The properties of COFs can be tailored by selecting different building blocks. Thus, they feature a large surface area and tunable pore size. The rigidity and discrete bond orientations of aromatic compounds make aromatic π‐systems an effective basis for COFs.^[^ ^130^ ^]^ The diverse aromatic systems allow for multiple combinations of building blocks, providing COFs with a high degree of flexibility in molecular design. Furthermore, this flexibility can enhance their piezoelectric performance. The piezoelectricity of 2D COFs composed of C_9_H_3_N_3_ and C_6_H_3_B_3_O_3_ monolayers has been studied through theoretical calculations (Figure 4f). Both monolayers exhibit hexagonal structures with space group *P*6, indicating non‐centrosymmetric crystal cells. When strain engineering is applied, the flexible structures of these materials can deform, leading to modulation of their electronic structures. The piezoelectric coefficient can be determined using the slope of a linear fit, which describes changes in clamping and relaxation polarization when uniaxial strain is applied in the direction of the armchair by the C_9_H_3_N_3_ and C_6_H_3_B_3_O_3_ monolayers. The calculated piezoelectric coefficients *d*_11_ are 0.93 pm V^−1^ for C_9_H_3_N_3_ and 0.83 pm V^−1^ for C_6_H_3_B_3_O_3_. These values are lower than those for inorganic piezoelectric materials, such as 2H‐MoS_2_ and 2H‐WS_2_. However, these COFs offer low cost, high flexibility, and scalability as catalysts, contributing positively to improve piezoelectric performance.^[^ ^131^ ^]^ The experimentally verified piezoelectricity of 2D COFs was recently demonstrated by Gu et al. (Figure 2g).^[^ ^132^ ^]^ Two distinct 2D COFs, designated as non‐centrosymmetric City‐U13 and City‐U14, were synthesized through the reaction of ^13^F‐CHO with 1,3,5‐tris(4‐aminophenyl)benzene and tris(4‐aminophenyl)triazine, respectively. These COFs demonstrated remarkably high piezoelectric properties, with *d*_33_ piezoelectric coefficients measured at 20.9 and 18.9 pC N^−1^, respectively.

### 2.4 GDY

Graphdiyne (GDY) is an emerging 2D material distinguished by its unique diacetylene structure, characterized by both *sp* and *sp* ^2^ hybridization. This configuration imparts high superior conductivity (10^−4^ to 10^−5^ cm^2^ V^−1^ s^−1^) and semiconducting properties, defined by a narrow bandgap (0.46–1.10 eV), which enables effective catalytic activity.^[^ ^133^, ^134^ ^]^ As illustrated in Figure 4h, GDY contains adjacent diacetylene linkages between two benzene rings, where *sp* and *sp* ^2^ hybridized carbon atoms are linked by “─C≡C─” units. These acetylenic linkages enhance the material's flexibility and fracture strain. Furthermore, the distribution of electron density in GDY is intrinsically uneven, leading to the emergence of a polarization electric field.^[^ ^135^ ^]^ The interconnected and uniformly hybridized networks exhibit large π‐conjugation and triangular porous backbones, providing numerous active sites that facilitate close contact between GDY and external molecules. This promotes efficient electron transfer to active sites, thereby improving catalytic activity.^[^ ^136^ ^]^ Moreover, due to its unique *sp* and *sp* ^2^ hybridization, the carrier mobility can reach 2.516 × 10^5^ cm^2^ V^−1^ s^−1^. The distinctive π‐conjugated structure offers a large surface area, uniform pores, excellent stability, hydrophobicity, charge carrier mobility, and low aggregation, demonstrating the significant potential of GDY in catalytic applications.^[^ ^137^ ^]^ Additionally, the inherent flexibility and stability of GDY facilitate modifications of its surface or internal structure.

Planar GDY belongs to the *P*_6_mm space group, with optimized lattice constants of *a* = *b* = 9.48 Å, and is thus classified as a non‐centrosymmetric 2D material.^[^ ^135^, ^138^ ^]^ Theoretical calculations and experimental studies have shown that GDY undergoes irregular deformation under mechanical stress, leading to the inconsistencies between internal positive and negative charge centers. This results in the formation of piezoelectricity.^[^ ^139^ ^]^ Furthermore, the alkene–alkyne complex transition effect of GDY significantly enhances its piezoelectric performance when combined with other piezoelectric materials (e.g., PVDF and BaTiO_3_) under ultrasonic activation. The reversible conversion from C≡C to C═C, with C≡C acting as an electron donor that releases electrons when a mechanical force is applied,^[^ ^65^ ^]^ contributes to the formation of a polar electric field due to dimensional changes in electron density around the alkyne bond. Consequently, GDY‐based heterojunctions exhibit enhanced piezoelectric activity.

Given the versatile and highly tunable structural properties of GDY, introducing external atoms to modulate electron cloud density represents a promising strategy for enhancing its performance. This approach not only disrupts structural symmetry through electron redistribution but also creates numerous active sites that amplify piezoelectricity. For instance, 4,12,2‐graphyne with a *P*4mm space group can be effectively doped with boron (B)/N atoms due to their facile modulating properties.^[^ ^140^ ^]^ N atoms, being electron‐rich, preferentially substitute *sp* sites due to their higher stability compared to doping at the *sp* ^2^ sites, while B atoms are introduced at the *sp* ^2^ sites. Their incorporation introduces charged electrons that significantly affect the distribution of covalent electrons around C atoms, weakening covalent bonds. This uneven distribution of electrons leads to the formation of local dipole moments. Thus, piezoelectricity can be generated in N/B‐doped GDY. Theoretical calculations indicate that the highest piezoelectric constants for B‐GDY and N‐GDY are 3.30 × 10^−10^ and 14.84 × 10^−10^ C m^−1^, respectively, which outperform those of 2D *h*‐BN, GaSe and GeSe. Similarly, introducing dipole pairs (e.g., CN^−^ and NH_2_^−^ groups) can induce an in‐plane dipolar electric field, and breaking the center symmetry of these dipole pairs generates additional piezoelectricity.^[^ ^141^ ^]^

Zhang et al. synthesized a highly electronegative S‐doped GDY where S was randomly incorporated into the benzene ring of the dialkyne unit to form C─S and C═S bonds (Figure 4i).^[^ ^142^ ^]^ The increased electron density may be localized in adjacent aromatic rings connected to S atoms due to the cooperative activation of electron‐rich S atoms and bonded C atoms. The S atoms disrupt the inversion symmetry of GDY, thereby inducing a piezoelectric effect. Additionally, the insertion of S atoms introduces impurity levels into the GDY network, reducing the energy gap and facilitating faster electron transfer into the CB.

### 2.5 Graphene and Its Derivatives

Graphene consists of 2D *sp* ^2^‐hybridized carbon, where C atoms form three strong covalent *σ* bonds in a hexagonal structure, with vertical orbitals contributing to planar π‐bonds.^[^ ^143^ ^]^ Its unique properties, such as high mechanical strength, excellent electrical conductivity, and large specific surface area, support the use of graphene in various electrical and optical applications.^[^ ^144^ ^]^ It has long been thought that intrinsic graphene exhibits no piezoelectric effect. This is primarily because bulk materials lacking a bandgap cannot demonstrate piezoelectricity, as conductors are unable to produce sufficient electric polarization. However, flexoelectricity has been used to explain the piezoelectric effect observed in some functionalized graphene and graphene nanoribbons.

Several strategies have been reported to achieve piezoelectric effects in graphene. Biaxial strain exerts pressure on graphene, causing deformation that alters its electrical properties.^[^ ^145^ ^]^ Specifically, applying atomic force microscopy (AFM) to measure the effect of pressure on surface potential reveals that as the back gate voltage increases, the corresponding surface potential rises. This occurs because the applied voltage bends the graphene, affecting its electronic structure and creating potential differences across cavity boundaries. Consequently, this potential difference promotes the separation of charge carriers, resulting in a band piezoelectric effect. The generated piezoelectric coefficient has been measured at 37 nC N^−1^. It should be noted that piezoelectric effects typically appear in insulators due to the very short shielding length of conductive metallic materials, which weakens polarization. In contrast, piezoelectricity in semi‐metallic graphene arises because the low carrier density in suspended graphene results in a longer shielding length, ensuring a significant voltage drop along the material. Similarly, first‐principal calculations demonstrate that penta‐graphene monolayers (CCC) and Janus penta‐graphene (CBB) exhibit out‐of‐plane piezoelectricity under stress or strain. The calculated piezoelectric coefficients *𝑑*_36_ are −0.065 pm V^−1^ for CCC and −0.418 pm V^−1^ for CBB, respectively.^[^ ^146^ ^]^ Atoms adsorbed on the graphene surface break inversion symmetry, hence inducing piezoelectricity. Furthermore, surface modification can adjust the piezoelectric properties of penta‐graphene (PG). Fluorinated PG, hydrofluorinated PG, and hydrogenated PG reduce the symmetry of PG. Hydrofluorination can introduce polarization between hydrogen (H) and fluorine (F) atoms and induce a transformation of graphene from a perfect regular hexagonal structure (centrosymmetry) to a zigzag shape (non‐centrosymmetry).^[^ ^147^ ^]^ It has been reported that a uniform coverage of C_2_HF, C_4_HF, Li, K, H, and F atoms can induce piezoelectricity by affecting electron distribution and altering the space group (Figure 4j).^[^ ^148^, ^149^ ^]^ Both Li and K atoms cause modest changes in piezoelectricity, adding F to the top site (on the C atom) and Li to the hollow site on the opposite side yields a maximum value for *d*_31_ of 0.3 pm V^−1^. Quantum mechanical calculations indicate that an in‐plane (*e*_11_) piezoelectric effect can be induced by designing defined triangular holes in monolayer graphene. Chandratre et al. designed a graphene sheet containing triangular holes that created a non‐centrosymmetric structure, generating significant polarization under mechanical force. Its piezoelectric coefficient is 0.124 C m^−2^.^[^ ^39^ ^]^ The piezoelectricity of graphene oxide was also investigated through DFT calculations, yielding a coefficient of 0.24 pm V^−1^, where deformation in the O‐doped region dominates the piezoelectric strain of the clamped graphene oxide (GO).^[^ ^150^ ^]^

### 2.6 CNTs

Carbon nanotubes (CNTs) are allotropes of carbon that belong to the fullerene structural family. They have diameters in the nanometer range and lengths in the micrometer range. Typical CNTs feature a tubular arrangement of hexagonal carbon atoms, which confer unique properties due to their symmetrical structure, including a high aspect ratio, excellent electrical conductivity, high melting point, low density (1.2–2.6 g cm^−3^), larger surface area (≈1000 m^2^ g^−1^), and remarkable flexibility.^[^ ^151^, ^152^, ^153^ ^]^ CNTs are classified into three types based on their synthesis methods: single‐walled carbon nanotubes, double‐walled carbon nanotubes, and multi‐walled carbon nanotubes (MWCNTs). Among these, MWCNTs have been demonstrated to possess piezoelectric properties, as evidenced by AFM tests (Figure 4k).^[^ ^154^ ^]^ During the tensile deformation process under a mechanical force, positive charge carriers concentrate at the top of the nanotubes, while the internal electric field strength opposes the direction of deformation, thereby inducing piezoelectricity. The piezoelectric coefficient could be measured at 0.107 ± 0.032 C m^−2^. Additionally, defects and N‐doping can significantly influence the piezoelectric properties of CNTs. CNTs synthesized via plasma‐enhanced chemical vapor deposition (CVD) often contain various defects. A low concentration of these defects can substantially enhance the piezoelectricity due to the redistribution of electron clouds.^[^ ^155^ ^]^ The introduction of N atoms can generate bamboo‐like defects resulting from the presence of pyrrole N, which are major contributors to the piezoelectric properties of N‐doped CNTs. Furthermore, the inserted N acts as a donor impurity that modifies the electronic band structure, leading to enhanced piezoelectricity in N‐doped CNTs compared to pristine CNTs.

## 3 Piezoelectric Mechanisms

The piezoelectric mechanism is a fundamental principle in materials science and engineering, with significant implications across various fields. At its core, piezoelectricity refers to the ability of certain materials to generate an electric charge in response to an applied mechanical stress.

### 3.1 Mechanisms of Piezoelectric Catalysis

**Figure 5:**
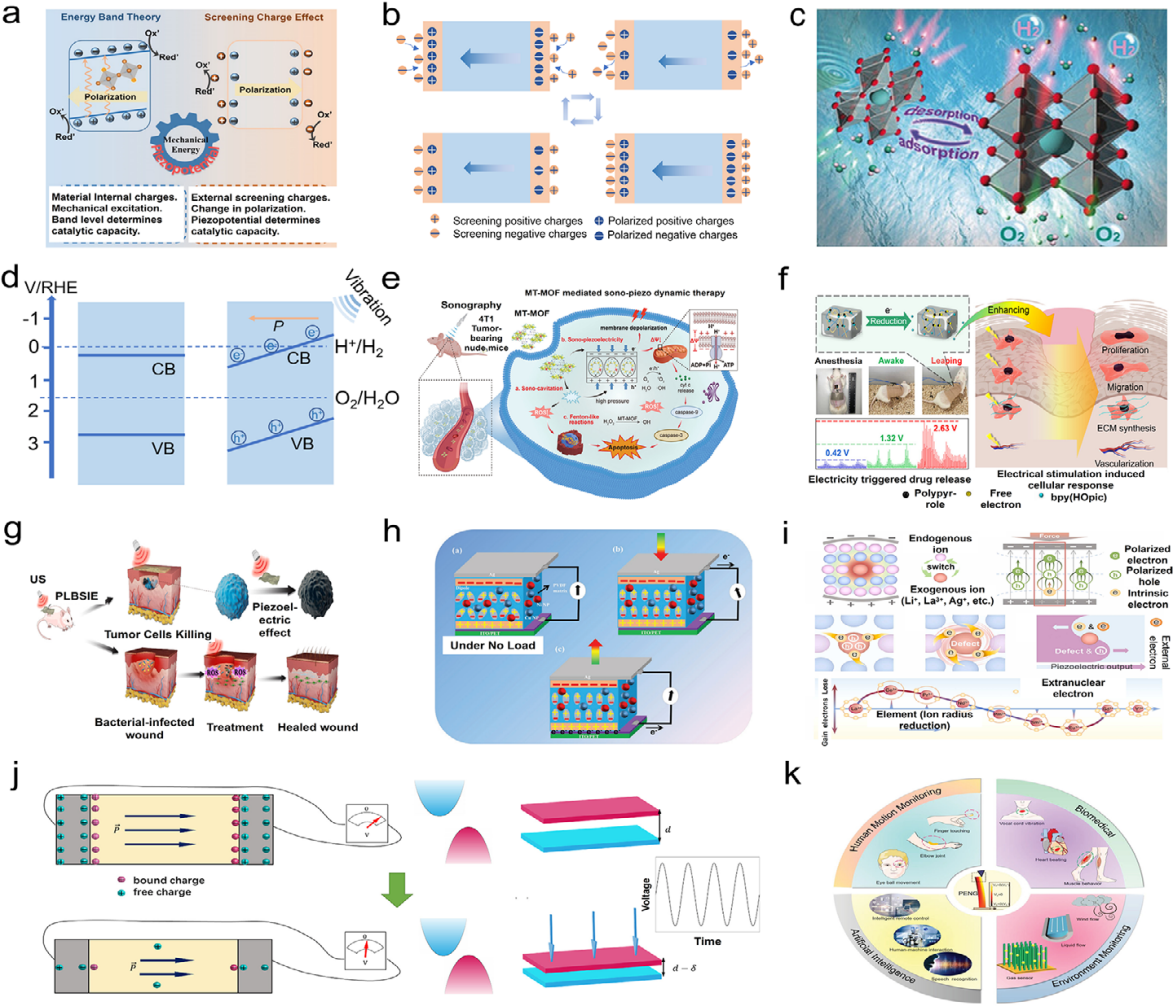
a) Mechanism of piezoelectric catalysis. Reproduced with permission.^[^ ^157^ ^]^ Copyright 2021 Wiley‐VCH. b) Schematic illustration of the screening effect in piezoelectric materials. c) H_2_ generation via the screening effect. Reproduced with permission.^[^ ^163^ ^]^ Copyright 2019, Wiley‐VCH. d) Diagram illustrating the energy band theory in piezoelectric catalysis. e) Piezoelectrically generated ROS for tumor treatment. Reproduced with permission.^[^ ^165^ ^]^ Copyright 2023, Wiley‐VCH. f) Piezoelectric‐induced electric stimulation for nerve repair. Reproduced with permission. Copyright 2023, American Chemical Society.^[^ ^166^ ^]^ g) The piezoelectric effect and ROS generation for anti‐infection, antitumor therapy, and wound healing. Reproduced with permission.^[^ ^168^ ^]^ Copyright 2023, American Chemical Society. h) The current generation process under external stress in a piezoelectric nanogenerator. Reproduced with permission.^[^ ^169^ ^]^ Copyright 2022, Wiley‐VCH. i) Enhanced piezoelectric polarization through the reduction of the screening effect. Reproduced with permission.^[^ ^173^ ^]^ Copyright 2022, Elsevier. j) Alternating generation of voltage under applied stress in a piezoelectric material. Reproduced with permission.^[^ ^174^ ^]^ Copyright 2023, American Chemical Society. k) Multifunctional applications of nanogenerators. Reproduced with permission.^[^ ^175^ ^]^ Copyright 2021, Wiley‐VCH.

In piezoelectric catalysis, materials with stronger piezoelectric properties exhibit higher catalytic activity. The mechanisms of piezoelectric catalysis are generally understood through the shielding effect and energy band theory (**Figure** **5**a).^[^ ^157^, ^158^ ^]^ The shielding theory posits that shielding charges, including internal carriers and external charges within the piezoelectric material, neutralize the piezoelectric charge, thereby facilitating the separation of electron–hole pairs. Specifically, the external shielding charges adsorbed on the surface of the piezoelectric material balance the polarization charges under ultrasonic irradiation. The induced enhancement of piezoelectric potential allows these external charges to be transferred to polarized charge sites with opposite electronegativities. When the applied force decreases, the piezoelectric field weakens, and the adsorbed external charges are released (Figure 5b). Desorbed charges with sufficient energy can promote redox reactions with differently charged molecules to form ROS, such as hydroxyl radicals (^•^OH), superoxide (^•^O_2_^−^), and hydrogen peroxide (H_2_O_2_). The piezoelectric potential plays a dominant role in the screening effect, analogous to the onset potential in electrocatalysis. For example, the redox potential of O_2_/H_2_O is 1.23 eV versus the reversible hydrogen electrode (RHE). Thus, the amplitude of the piezopotential must fully reach or exceed the Gibbs free energy threshold required for water splitting to effectively facilitate these reactions. Taking typical BaTiO_3_ as an example, a nanowire with a diameter of 10 nm can exhibit an excellent piezoelectric coefficient of 0.45 pm V^−1^, while its calculated piezoelectric potential reaches 2.6 V. Under periodic vibration, this 10 nm nanowire could release active species with high energy, which split water into H_2_ and O_2_. (Figure 5c).^[^ ^159^ ^]^ The relationship between piezoelectric potential (*P*) and screening charge density (*σ*_s_) can be expressed by the following equations:^[^ ^160^, ^161^ ^]^

(1) $$\sigma_{s}=d\cdot \frac{P}{\left[\varepsilon_{r}\left(\delta_{1}+\delta_{2}\right)+d\right]}$$

(2) $$V=\left(\frac{d_{ij}}{\varepsilon_{r}\varepsilon_{0}}\right)\;\sigma w$$

where *d* and ε_r_ represent thickness and relative permittivity, respectively. δ_1_ and δ_2_ are the thicknesses of the screening charge layers on two polar surfaces. *V* is the piezopotential. *d_ij_* is the piezoelectric coefficient. ε_0_ is the electrical permittivity of free space. σ represents the loading of stress, and *w* is the width of the piezoelectric particle. Based on these equations, a stronger external force can generate a higher piezopotential, leading to an increased screening charge density. Su et al. reported that strain‐engineered BaTiO_3_ achieved an enhancement of piezopotential to 1.6 V through finite element method calculations, enabling it to produce H_2_ and O_2_. Therefore, engineering sufficiently high piezopotentials is crucial for making surface mobile charges thermodynamically favorable for reactions.^[^ ^162^ ^]^

The energy band theory in piezoelectric catalysis is analogous to photocatalytic mechanisms, wherein electrons are excited and transferred from VB to CB. Subsequently, electron–hole pairs are further separated and transported to the surface to participate in redox reactions. The energy band structure and electronic states of piezocatalysts are critical factors that determine their catalytic activities. Ultrasound‐induced cavitation bubbles generate significant energy and pressure for electron excitation when piezoelectric materials are subjected to ultrasound. Under static strain conditions, the generated piezoelectric field can induce tilting of the energy band. However, this effect may be shielded by oriented accumulation of internal carriers. Periodic vibrations formed by ultrasound provide fluctuations for the piezoelectric potential field, causing internal carriers to remain in a metastable state rather than accumulating in a specific orientation. Energy band tilting contributes to an increase in redox potentials for both VB and CB, thereby enhancing the redox activity (Figure 5d). For instance, catalytic production of H_2_ (0 vs RHE) on BiFeO_3_ is thermodynamically unfavorable because its band levels do not reach the redox potentials required for water splitting. However, applying strain to BiFeO_3_ generates a significant increase in piezopotential up to 0.88 V, which is sufficient to tilt its band structure and align CB positions with reduction potential levels. This rearrangement of bands is energetically favorable for H_2_ evolution.^[^ ^163^ ^]^ Meanwhile, the piezoelectric field provides a driving force that accelerates carrier transport and separation by reducing electronic conduction resistance. This can be confirmed by observing enhanced piezoelectric current and reduced electrochemical impedance spectra with increasing ultrasonic power for SnS.^[^ ^164^ ^]^

During the catalytic process, stability is a crucial factor for achieving long‐term catalytic activity. Carbon‐based materials are known for their excellent chemical stability, mechanical durability, and thermal stability. Their resistance to oxidation and chemical degradation makes them suitable for use in harsh environments. For instance, Li et al. demonstrated that PVDF could maintain stability and catalytic activity under strong acid and high temperature conditions. Forming composites with other materials can further enhance their stability and durability. For example, reduced GO (rGO)/PVDF maintained its structural integrity and catalytic activity in strong acidic (pH = 1) and basic (pH = 13) conditions and showed good stability under a slightly elevated temperature of 40 °C. Under these extreme conditions, rGO/PVDF could still achieve more than 78% removal efficiency of pollutants through piezoelectric catalysis, which is better than that of the pristine PVDF film, highlighting its potential for stable catalytic performance.^[^ ^35^ ^]^ Zeng et al. investigated the fatigue resistance of CNT‐based composites, finding that they maintained their piezoelectric properties after repeated mechanical cycling.^[^ ^36^ ^]^

### 3.2 Mechanism of Piezoelectric Biomedical Treatment

Piezoelectric biomedical treatments are generally achieved by combining piezoelectric biomaterials with ultrasound, leveraging the biocompatibility and non‐toxicity of biomaterials alongside the non‐invasive and penetrating capabilities of ultrasound. Two widely recognized mechanisms are ROS generation and electric stimulation (ES). In the ROS mechanism, piezoelectric biomaterials with excellent biocompatibility enter cells through the cell membrane. Under vibration, these biomaterials deform and induce a piezoelectric field, leading to the generation of ROS, such as ^•^OH, ^•^O_2_^−^, H_2_O_2_, and singlet oxygen (^1^O_2_). The oxidative ROS can damage cellular components, which include induction of tissue necrosis and DNA damage, ultimately resulting in cell apoptosis. This strategy is often employed to kill tumor cells and combat infections. For example, in Figure 5e, Mn─Ti bimetallic organic framework tetragonal nanosheets (MT‐MOF TNS) exhibit good biocompatibility with 4T1 tumor cells and are engulfed by them. Ultrasound cavitation induces MT‐MOF TNS to generate ROS and local charges piezoelectrically. This process can cause severe mitochondrial dysfunction and activate the mitochondrial apoptosis signaling pathway, leading to the apoptosis of tumor cells.^[^ ^165^ ^]^ In contrast to the ROS mechanism, ES originates from piezoelectric voltage. Externally induced piezoelectricity can create a local electric field that provides a microenvironment for stimulating cells through electron transfer. This stimulation affects biological signaling pathways and promotes the production of desired effector molecules that aid in cell repair with high biosafety. For instance, when a rat with wounds is equipped with a wearable P(VDF‐TrFE) device and engages in motions, the vibration‐induced piezoelectric voltage benefits wound healing due to accelerated electron transfer. Additionally, more strenuous exercise (e.g., leaping) could generate higher intensity of voltage that contributes to the release of drug, thereby improving tissue repair (Figure 5f).^[^ ^166^ ^]^ The piezoelectric effect of rGO@UIO‐66/polycaprolactone (PCL) scaffolds can enhance the expression of K_ATP_ channels and reduce Ca^2+^ influx in macrophages, thereby supporting nerve repair.^[^ ^167^ ^]^ A piezoelectric elastomer was constructed by copolymerizing with lactic acid, butanediol, sebacic acid, and itaconic acid. The stretch recovery curve of this elastomer demonstrates full recoverability after 500 loading and unloading cycles, with a residual polarization *P*_r_ value of 1.6 mC m^−2^ obtained under a coercive electric field of 13 MV m^−1^, indicating excellent stretchability and piezoelectric properties. The piezoelectric elastomer was applied to wounds infected with *Staphylococcus aureus* (*S. aureus*) in mice and in an MNNG/HOS tumor‐bearing mouse model. When ultrasound was activated for several days, the wounds healed, and tumors were eradicated. The destruction of *S. aureus* and MNNG/HOS tumors is attributed to the ROS generated by the piezoelectric effect. During wound healing, cell migration was observed. This is attributed to the piezoelectric effect inducing appropriate ROS production, which promotes cell migration by regulating the local cytoskeleton at the molecular level through the initiation of redox signaling cascades (Figure 5g).^[^ ^168^ ^]^

### 3.3 Mechanism of Piezoelectric Nanogenerators

Piezoelectric energy harvesting technology involves converting mechanical energy into electrical energy. Piezoelectric nanogenerators (PENGs) are typically composed of piezoelectric materials sandwiched between two electrodes positioned at the top and the bottom. Regarding the generation of piezoelectric current, when strain or compressive stress is applied to the piezoelectric device, a piezopotential gradually forms, and polarization changes occur, creating a negative charge on one side of the material and a positive charge on another side. The arrangement of electric dipoles in a specific direction produces significant piezoelectric potential. At this point, if electrodes, such as conductive fabric, are connected to external conductive wires, charge will flow from one electrode to the other, thereby generating an output voltage and a current signal. The electrostatic potential generated by the polarization charges is balanced by the flow of electrons from one electrode to the other through an external load. When the stress is removed, the piezoelectric effect weakens, and the charge accumulated on the electrode side moves in the opposite direction, generating a reverse electrical signal (Figure 5h).^[^ ^169^ ^]^ The relationship between output current, applied strain, and polarization charges is represented by the following equations:^[^ ^170^, ^171^ ^]^

(3)

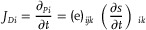

(4) $$J_{Dz}=\frac{\partial_{Pz}}{\partial t}=\frac{\partial \sigma_{P}\left(Z\right)}{\partial t}\;$$

where *J_Di_* and *J_Dz_* are the output currents, *s* is the applied strain, (e)_*ijk*_ is the piezoelectric tensor, and σ_P_(*Z*) represents surface polarization charges along the *z*‐axis. Therefore, increasing the frequency of an applied strain and altering the rate of polarization contributes to the improved output current. The open‐circuit voltage for PENGs can be represented by the equation:^[^ ^172^ ^]^

(5) $$V_{\mathrm{oc}}=z\sigma_{P}\left(Z\right)/\in$$

Clearly, increasing surface polarization charge density can enhance open‐circuit voltage.

For PENGs, the shielding effect exerts a negative influence on piezoelectricity because piezoelectric charges can be neutralized by screening charges. The internal screening effect can be mitigated by accelerating the recombination of holes and electrons, as well as improving carrier mobility. Doping with rare earth ions induces defects and introduces holes that effectively capture electrons, thereby enhancing piezoelectricity. Additionally, variations in ionic radius and extranuclear electron count can have diverse effects on piezoelectric polarization (Figure 5i).^[^ ^173^ ^]^ Specifically, Y^3+^ ions with a small radius and eight extranuclear electrons doped into ZnO can achieve an output voltage of ≈2.5 V, outperforming other rare earth ions doped into ZnO. Furthermore, increasing applied force intensity can lead to higher polarization charge density. For 2D piezoelectric materials, applying pressure can induce a transition from an insulator to a metallic state by reducing the bandgap. This pressure also causes significant changes in in‐plane polarization, resulting in high piezoelectric efficiency. Consequently, a high alternating voltage can be generated (Figure 5j).^[^ ^174^ ^]^

PENGs serve as sustainable power sources for electronic devices and can also generate electrical signals that directly sense applied mechanical actions without requiring an external power supply. Flexible nanogenerators are widely developed because they can convert weak vibration energy, such as compression and bending, into electrical energy. Therefore, flexible PENGs can be designed as wearable devices for applications in human motion monitoring, biomedical treatment, artificial intelligence, and environmental monitoring (Figure 5k).^[^ ^175^ ^]^

### 3.4 Key Factors Affecting Piezoelectric Properties

#### 3.4.1 Structural Defects

When investigating the potential origins of charge carriers in piezocatalysis, it is essential to consider the intrinsic unpaired or free charges that arise from defects, as they play a significant role and cannot be overlooked. The piezopotential‐induced migration of free electrons to the material's surface leads to the formation of reactive species such as ^•^O_2_^−^ and H_2_O_2_. These reactive species play a crucial role in energy conversion, environmental treatment and biomedical applications. Common anion vacancies, such as those of oxygen, nitrogen, and carbon in carbon‐based materials, contribute to the generation of free electrons within the material, thereby maintaining charge neutrality.^[^ ^157^ ^]^ Consequently, defect engineering is actively pursued to increase the concentration of free charges, thereby enhancing the piezocatalytic activity. For instance, Zhang et al. demonstrated that introducing vacancy defects in microcrystalline cellulose can improve its piezoelectric response by increasing asymmetry under external force, which enhances the titling of energy bands and accelerates carrier transfer.^[^ ^176^ ^]^ S. K. Nevhal and S. I. Kundalwal demonstrated the mechanism of induced piezoelectricity in armchair graphene nanoribbons (AGNRs) systems with non‐centrosymmetric pores, as well as defects such as divacancies, line defects, and Stone–Wales defects, using first‐principle calculations.^[^ ^177^ ^]^ The introduction of defects in graphene nanoribbons (GNRs) results in the formation of bandgaps, altering their electronic structure and disrupting the centrosymmetry of the GNR systems. When axial force is applied, these defects cause bandgaps to emerge at the Fermi level in the defective GNRs, inducing strain gradient polarization as a consequence of the flexoelectric effect. Additionally, enhancing the defect concentration in AGNRs leads to an amplified piezoelectric effect, attributed to the increased alignment of dipole moments.

#### 3.4.2 Heteroatom Doping

Doping carbon‐based materials with heteroatoms like nitrogen or boron can modify their electronic structure and piezoelectric properties. Il'ina et al. have experimentally demonstrated that nitrogen‐doped CNTs exhibit piezoelectric properties, with the magnitude of this effect being dependent on the concentration of doped pyrrolic nitrogen.^[^ ^155^ ^]^ They propose a mechanism for the emergence of a piezoelectric response in CNTs, which is linked to the formation of an uncompensated dipole moment. This arises from the curvature of the sheet surface, induced by the presence of pyrrolic‐like defects and bamboo‐like “bridges” within the nanotube cavity, indicating that as the pyrrolic nitrogen concentration increases, the piezoelectric coefficient rises from 30 to 92 pm V^−1^. Concurrently, the current generated during CNT deformation could increase to 129 nA. Chlorine (Cl)‐doped g‐C_3_N_4_ demonstrates a noticeable enhancement in transient piezocurrent compared to pristine g‐C_3_N_4_, consistent with its improved piezoelectric properties.^[^ ^178^ ^]^ This improvement indicates more efficient charge separation and transmission facilitated by the piezopotential field. This enhancement might be attributed to the creation of polarized bonds and changes in the electronic structure.

#### 3.4.3 Strain Engineering

A built‐in piezopotential field aligned with the dipole moment within piezoelectric materials can be effectively established through mechanical straining, leading to alterations in the bulk electronic state and resulting in band tilting. Unlike perfect insulators, where the band levels tilt linearly across the strained region due to the absence of mobile charges responding to the piezoelectric potential field, the band tilting in piezoelectric semiconductors can be mitigated by the oriented accumulation of internal mobile charges.^[^ ^45^ ^]^ Specifically, under static deformation, the piezopotential drives opposing mobile charges to continuously accumulate on the two polar sides, creating a depolarization field that counteracts the piezoelectric polarization effect and thus screens the band tilting.^[^ ^158^ ^]^ To address this, periodic mechanical strain is necessary in piezocatalysis to prevent the screening of band tilting. With periodic vibrations, such as those induced by ultrasound, the resulting fluctuations in the piezoelectric potential field maintain the internal mobile charges in a metastable state, preventing their oriented accumulation. Consequently, this approach prevents the screening of band tilting and allows for the manipulation of electronic energy levels in a piezoelectric semiconductor.^[^ ^145^ ^]^

One of the most significant advantages is the enhancement of redox capability. Even the catalytic generation of ^•^O_2_^−^ and ^•^OH radicals is thermodynamically unfavorable in some materials. Under periodic vibration, they could be produced due to band tilting that enhances redox potential. Applying mechanical strain to carbon‐based materials can induce changes in their band structure, thereby affecting their piezoelectric behavior. Li et al. reported a piezoelectric CQD‐C_3_N_5_ composite, which could generate 5025 µmol g^−1^ h^−1^ H_2_O_2_ under ultrasonic irradiation.^[^ ^179^ ^]^ To further investigate the impact of pressure on energy band, they applied in situ high‐pressure UV–vis spectroscopy. The bandgap decreases as the pressure increases. The piezoelectricity is relevant to the intensity of external pressure. Hence, pressure could promote the transfer of electrons.

## 4 Applications for Carbon‐Based Piezoelectric Materials

### 4.1 Piezoelectric Catalysis

Generally, higher redox potential and stronger redox abilities are desirable. However, a larger bandgap in semiconductors can be detrimental to the transfer of electrons from VB to CB. Hence, piezoelectric catalytic activity depends on the combined effects of redox potential and bandgap energy. To further enhance piezoelectric performance, several strategies have been employed, including exposing more active sites, heteroatom‐doping or anchoring, constructing heterostructures, and utilizing strain or light assistance.^[^ ^13^, ^57^, ^178^, ^179^, ^180^ ^]^ Specifically, surface modifications such as defects or vacancies can increase active sites and introduce impurity levels to improve mass transfer mass rates and reduce the bandgap. Heterostructures form interfaces with built‐in electric field that accelerate electron and hole mobility. As piezoelectric materials possess light absorption properties, the generated piezoelectric effect can efficiently improve the separation efficiency of photogenerated electron–hole pairs in both the bulk phase and at the surface, making it a viable method to enhance the photocatalytic ability.

#### 4.1.1 Environmental Remediation

Environmental pollution remains a serious issue globally as economies and industrialization progress rapidly. Contaminants such as bacteria, heavy metals, and chlorophenols produced by petrochemicals, pharmaceuticals, and synthetic dye industries pose significant risks.^[^ ^180^ ^]^ These contaminants exhibit potential carcinogenicity, teratogenicity, and biotoxicity, necessitating the development of efficient remediation methods. Piezoelectric catalysis has been extensively studied for wastewater treatment, demonstrating excellent catalytic activity and environmental compatibility with carbon‐based piezoelectric materials. ROS (i.e., ^•^O_2_^−^, ^•^OH, and ^1^O_2_) play a crucial role in pollutant removal.

**Figure 6:**
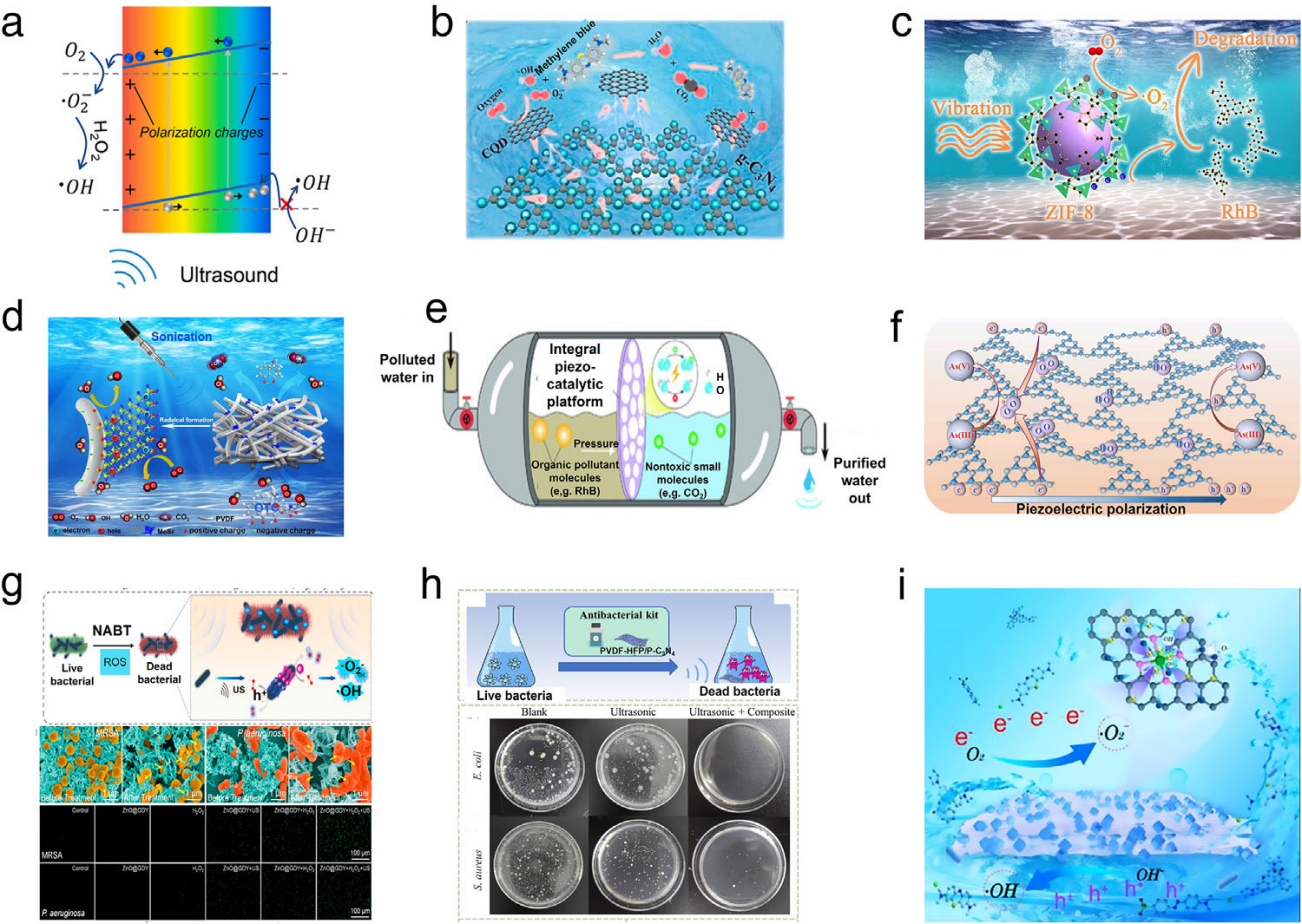
a) ROS generation by g‐C_3_N_4_ under ultrasound. Reproduced with permission.^[^ ^181^ ^]^ Copyright 2021, Elsevier. b) Degradation mechanism of MB for CQD/g‐C_3_N_4_ composites under ultrasound. Reproduced with permission.^[^ ^66^ ^]^ Copyright 2023, Elsevier. c) Degradation mechanism of Rh B for ZIF‐8 MOF under ultrasound. Reproduced with permission.^[^ ^123^ ^]^ Copyright 2022, Elsevier. d) Tetracycline degradation by PVDF/MoS_2_ membranes under ultrasound. Reproduced with permission.^[^ ^185^ ^]^ Copyright 2021, Elsevier. e) Conceptual illustration of water treatment using an integral piezocatalytic PVDF/BaTiO_3_ membrane. Reproduced with permission.^[^ ^186^ ^]^ Copyright 2021, The Authors, published by Wiley‐VCH. f) Removal of As(III) by piezoelectric catalysis in g‐C_3_N_4._ Reproduced with permission.^[^ ^190^ ^]^ Copyright 2021, Elsevier. g) Schematic diagram of the antibacterial mechanism of ZnO@GDY NRs. Reproduced with permission.^[^ ^191^ ^]^ Copyright 2022, American Chemical Society. h) Illustration of the antibacterial kit sterilization process using piezoelectric polymer composites. Reproduced with permission.^[^ ^192^ ^]^ Copyright 2022, Wiley‐VCH. i) Piezoelectric degradation and antibacterial mechanism of Ca‐CN. Reproduced with permission.^[^ ^194^ ^]^ Copyright 2024, American Chemical Society.

2D ultrathin g‐C_3_N_4_ nanosheets with an approximate thickness of 2 nm were synthesized using a modified thermal polycondensation approach. Under ultrasound excitation, 83.1% of 2,4‐dichlorophenol (20 mg L^−1^) could be removed, along with efficient removal of 2,5‐dichlorophenol, 2,6‐dichlorophenol, and 4‐chlorophenol. ^•^O_2_^−^ and ^•^OH are the major ROS responsible for attacking pollutant molecules.^[^ ^181^ ^]^ The rupture of cavitation bubbles generates immense stress (up to ≈10^8^ Pa) on the ultrathin 2D g‐C_3_N_4_. Thus, the deformation of 2D g‐C_3_N_4_ induces piezoelectric polarization due to its triangular non‐centrosymmetric pores in the host layer. The oppositely polarized charges create a piezoelectric field that causes tilting of the energy band structure, thereby promoting charge carrier transfer (**Figure** **6**a). Although the VB (1.3 eV vs NHE) is insufficient to directly oxidize OH^−^ to ^•^OH, the formation of ^•^OH occurs due to the decomposition of H_2_O_2_ generated by the reaction between ^•^O_2_^−^ with H_2_O. To further enhance the charge carrier mobility, Yan et al. deposited CQDs on g‐C_3_N_4_. Under ultrasonic vibration for 30 min, 92.5% methylene blue (MB) could be removed. The CQDs not only accelerate the transfer of free charges but also serve as active sites to promote the interactions between molecules and materials (Figure 6b).^[^ ^66^ ^]^ Considering the outstanding visible light absorption properties of g‐C_3_N_4_, combining photocatalysis with piezoelectric catalysis can further enhance the catalytic activity. The piezo‐photocatalytic efficiency for chlorophenols could reach ≈100% because light excitation facilitates greater electron transfer, leading to increased ROS generation. Moreover, the thickness and porosity of g‐C_3_N_4_ significantly affect its piezoelectric catalytic performance. Chen et al. reported an ammonium oxalate‐modified g‐C_3_N_4_ that exhibits an ultrathin nanosheet with a thickness of 1.52 nm. This material has a piezoelectric degradation rate constant for Rh B that is twice that of bulk g‐C_3_N_4_. Thin nanosheets can absorb more mechanical energy to generate a stronger piezoelectric field while exhibiting a higher N─(C)_3_ to C─N─H area ratio. Therefore, they possess more bonded tri‐*s*‐triazine units arranged orderly in layers to produce higher in‐plane piezoelectricity as polar groups.

Piezoelectric MOFs have also been applied to treat contaminants. ZIF‐8 nanoparticles achieved ≈94.5% removal of Rh B after undergoing 90 min of vibration. During this process, ^•^O_2_^−^ acts as the primary redox species responsible for decomposing Rh B (Figure 6c).^[^ ^123^ ^]^ Piezoelectric activation of persulfate has emerged as an effective strategy for pollutant degradation. This has been extensively investigated using inorganic piezoelectric materials like BaTiO_3_ and SrBi_2_B_2_O_7_,^[^ ^182^, ^183^ ^]^ although studies on organic piezoelectrics remain limited. Recently, metal–organic framework Bio‐MOF‐1 was shown to piezoelectrically activate peroxymonosulfate (PMS), generating singlet oxygen (^1^O_2_) as the major active oxygen species, which effectively removes 98.5% bisphenol A, a compound released from plastics that poses serious threats to human health.^[^ ^184^ ^]^ MIL‐100(Fe) achieved 92% removal of carbamazepine under ultrasonic cavitation. Mechanical force induces deformation within the MOF structure, creating a built‐in electric field that promotes free charge migration while modulating the band structure of the sample.^[^ ^121^ ^]^ The more positive oxidation potential compared to H_2_O_2_/OH and more negative reduction potential than ^•^O_2_^−^ are suitable for generating ROS to attack pollutants.

The piezoelectric catalytic activity of pure PVDF has been investigated in pollutant degradation. However, its performance is limited due to the low *β*‐phase piezoelectric content. Typically, PVDF is composed of other inorganic piezoelectrics to significantly improve its piezoelectricity for practical applications. It has been reported that pure PVDF could only remove 15.87% oxytetracycline (OTC) under ultrasonic conditions. However, incorporating MoS_2_ nanosheets greatly enhanced its degradation activity to remove 93.08% OTC (Figure 6d).^[^ ^185^ ^]^ MoS_2_ acts as a nucleating agent to increase *β*‐phase concentration while generating more active sites for interactions between the polymer matrix and pollutant molecules. This also enhances piezoelectric potential to accelerate charge carrier separation and transfer. Shi et al. synthesized PVDF/BaTiO_3_ composites where BaTiO_3_ nanoparticles are embedded within a PVDF scaffold. The *γ*‐phase content increased significantly while the piezoelectric catalytic degradation efficiency for Rh B improved from ≈30% to 87% when ultrasonic power was increased from 60 to 180 W. This enhancement of piezoelectric potential correlates with increasing ultrasonic power (Figure 6e).^[^ ^186^ ^]^ Similarly, constructing heterojunctions between inorganic piezoelectrics and PVDF is an effective method for achieving pollutant removal. For example, BaTiO_3_/HPVDF nanocomposite fibers exhibit excellent piezoelectric effects because BaTiO_3_ promotes *β*‐phase conversion.^[^ ^187^ ^]^ The flexible film can easily deform under external forces to induce the piezoelectric potential. Additionally, BaTiO_3_ can also generate a piezoelectric field. Together, their effects accelerate charge carrier transfer and separation while generating numerous radicals for pollutant removal. This phenomenon of enhanced piezoelectric effects through increased *β*‐phase content has also been investigated in ZnO/CQDs/PVDF composites.^[^ ^185^, ^188^ ^]^ Wang et al. reported that the bi‐piezoelectric effect enhances photocatalytic activity in ZnO/PVDF by forming a piezoelectric polarization field in both ZnO and PVDF under pressure. This significantly accelerates electron transfer and radical formation, which effectively degrades 95% methyl orange (MO) under stirring at 1000 rpm with light irradiation.^[^ ^189^ ^]^

As(III), known for its acute toxicity and carcinogenicity, is widely present in water environments. Utilizing piezoelectric catalysis can transform As(III) into less toxic As(V). Shu et al. reported that polymeric carbon nitride (PCN) and crystalline carbon nitride (K‐PHI) efficiently oxidize As(III) to As(V) under ultrasonic conditions. K‐PHI, with its higher surface potential and stronger piezoelectric properties, demonstrated superior oxidation ability by oxidizing 98.9% As(III), achieving an oxidation rate constant of 1.486 h^−1^, 6.72 times greater than that of PCN.^[^ ^190^ ^]^ The accelerated separation and transfer of electrons enable them to react with O_2_ to form ^•^O_2_^−^, while h^+^ act as the oxidative species. Both contribute to oxidizing As(III) into As(V) (Figure 6f).

Combining piezoelectric catalysis with H_2_O_2_ or persulfate forms a Fenton‐like system where electrons generated from piezoelectric effects activate oxidative agents to produce higher concentrations of ROS, thus enabling efficient decomposition of refractory materials and bacterial. ZnO@GDY combined with H_2_O_2_ can piezoelectrically inactivate methicillin‐resistant *Staphylococcus aureus* at concentrations of around 35 ± 1 µg mL^−1^ and *Pseudomonas aeruginosa* (*P. aeruginosa*) at concentrations of around 53 ± 1 µg mL^−1^ under ultrasound conditions. ZnO@GDY deforms to induce a piezoelectric field that drives charge carrier transfer into surfaces where they can electrochemically react with H_2_O and O_2_, while activating H_2_O_2_ into ROS (^•^OH, ^•^O_2_^−^). This process disrupts bacterial biofilms leading to sterilization (Figure 6g).^[^ ^191^ ^]^ Chen et al. developed a piezoelectric polymer composite comprising a matrix PVDF‐HFP combined with P‐doped C_3_N_5_. Under ultrasonic action, these composites effectively reduce O_2_ into ^•^O_2_^−^ and H_2_O_2_. These reactive species can effectively inactivate *E. coli* and *S. aureus* bacteria (Figure 6h).^[^ ^192^ ^]^ Ag@LiNbO_3_/PVDF composite films achieved ≈100% removal of *Escherichia coli* and 96.65% removal of *Staphylococcus aureus* within 180 min under ultrasound conditions. LiNbO_3_ not only provides piezoelectric potential but also improves *β*‐phase content in PVDF. Ag further enhances hole–electron separation and transfer rates, resulting in substantial ROS generation necessary for bacteria inactivation.^[^ ^193^ ^]^ In catalysis research fields, single atoms have been adopted to achieve near‐complete conversion efficiency. Zhao et al. synthesized a composite featuring Ca atoms embedded within N‐doped C alongside PVDF, demonstrating a record‐high degradation rate constant of 0.11 min^−1^ for Rh B along with an antibacterial efficiency of 99.8% against *E. coli*, in which ^•^O_2_^−^ and ^•^OH are dominant active species. Contact angle studies combined with DFT calculations confirm that Ca not only provides sufficient active sites along with local polarized electric fields but also enhances both hydrophilicity and piezoelectricity within PVDF (Figure 6i).^[^ ^194^ ^]^ It has also been reported that S‐doped GDY acts as an enzyme mimic by activating H_2_O_2_ through piezoelectric means, generating substantial ROS capable of inactivating both Gram‐positive *S. aureus* strains as well as Gram‐negative *E. coli* strains under ultrasound exposure.^[^ ^142^ ^]^

#### 4.1.2 Hydrogen Evolution

Hydrogen (H_2_) is considered an important alternative to traditional energy sources due to its high energy density and environmental friendliness. Piezoelectric H_2_ evolution has emerged as a promising strategy, involving several processes. Mechanical stress is applied to the piezoelectric material through various methods, such as ultrasound and fluid flow. The applied stress causes the piezoelectric material to deform, generating an electric field within the material due to the displacement of positive and negative charges. The resulting electric field drives the electrochemical reaction of water splitting, which involves breaking down water molecules into H_2_ and O_2_ gases. Protons (H^+^) in the water are attracted to the negatively charged side of the piezoelectric material, while electrons are attracted to the positively charged side. On the negatively charged side, protons gain electrons (reduction reaction) to form H_2_ gas. Simultaneously, at the positively charged side, water molecules lose electrons (oxidation reaction) to produce O_2_ gas and protons.^[^ ^33^ ^]^ Furthermore, the abundant hydrogen bonds between the polymer unit chains promote the adsorption and activation of H_2_O molecules, suggesting that carbonyl polymers may serve as ideal materials for harvesting mechanical energy to evolve H_2_.

**Figure 7:**
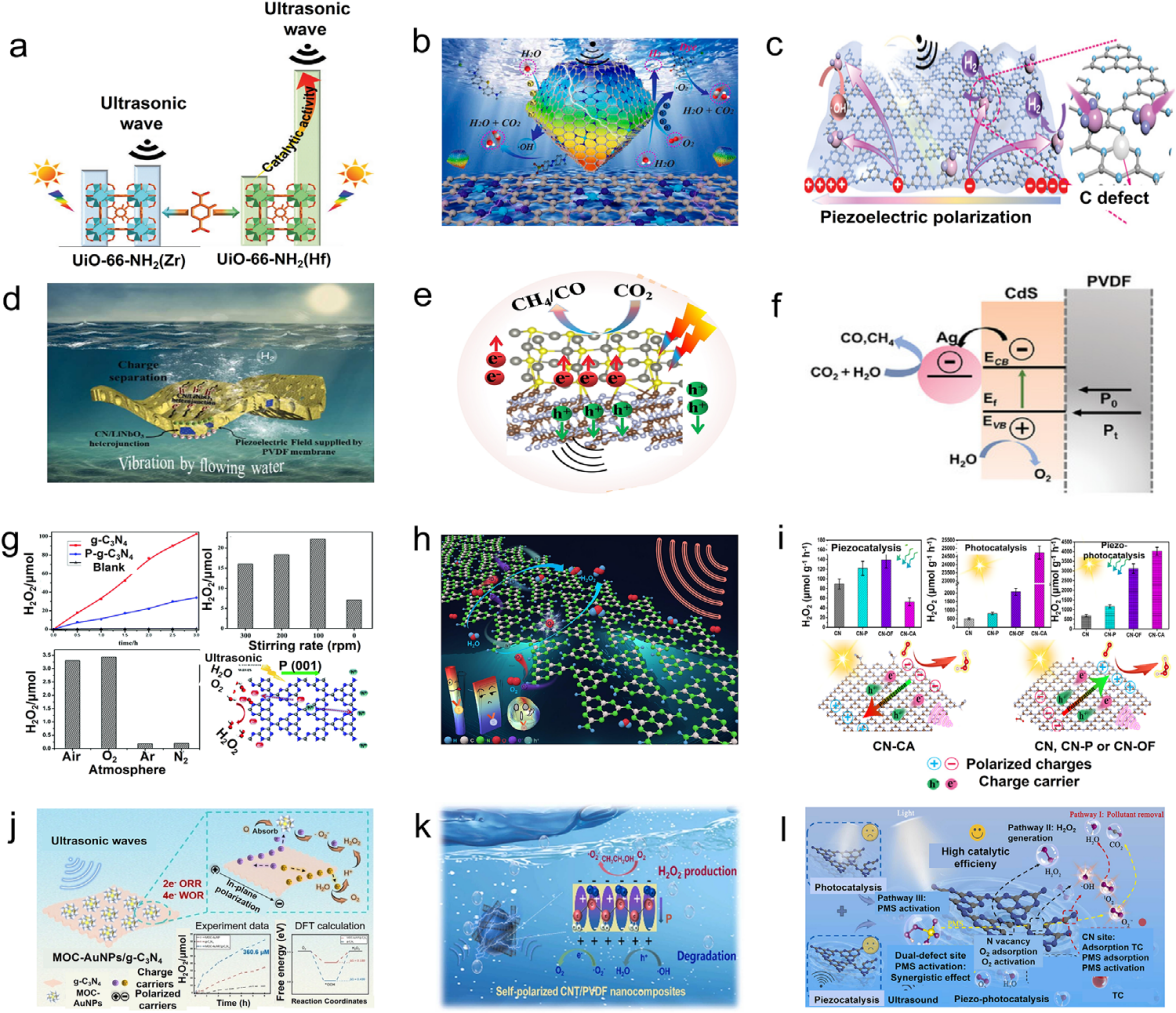
a) Schematic representation of H_2_ production activities of UiO‐66‐NH_2_ (Zr) and UiO‐66‐NH_2_ (Hf) in piezo‐photocatalysis. Reproduced with permission.^[^ ^124^ ^]^ Copyright 2021, Wiley‐VCH. b) Schematic illustration of the piezocatalytic dye degradation and H_2_ production over the Zn─N*_x_* ─C piezocatalyst. Reproduced with permission.^[^ ^120^ ^]^ Copyright 2023, Elsevier. c) Schematic illustration of the photo‐piezocatalytic process over UT‐g‐C_3_N_4_. Reproduced with permission.^[^ ^48^ ^]^ Copyright 2021, Wiley‐VCH. d) Scheme depicting piezocatalytic H_2_ production over the g‐C_3_N_4_/LiNbO_3_/PVDF piezocatalytic membrane under water flow conditions. Reproduced with permission.^[^ ^55^ ^]^ Copyright 2022, Elsevier. e) Piezoelectric conversion of CO_2_ into CO and CH_4_ over ZnS/g‐C_3_N_4_. Reproduced with permission.^[^ ^197^ ^]^ Copyright 2022, Wiley‐VCH. f) Mechanism of piezoelectric reduction of CO_2_ over Ag/CdS/PVDF. Reproduced with permission.^[^ ^81^ ^]^ Copyright 2023, Wiley‐VCH. g) Piezoelectric generation of H_2_O_2_ over g‐C_3_N_4_. Reproduced with permission.^[^ ^200^ ^]^ Copyright 2023, The Royal Society of Chemistry. h) The effect of g‐C_3_N_4_ morphology on piezoelectric H_2_O_2_ generation. Reproduced with permission.^[^ ^54^ ^]^ Copyright 2023, Elsevier. i) Enhanced piezoelectric photocatalytic H_2_O_2_ generation over phosphorus‐modified (CN‐P), oxygen‐functionalized CN (CN‐OF), and cyano‐group grafted. Reproduced with permission g‐C_3_N_4_. Reproduced with permission.^[^ ^201^ ^]^ Copyright 2023, American Chemical Society. j) Mechanism for accelerating electron transfer and O_2_ adsorption to generate H_2_O_2_ over MOC‐AuNPs/g‐C_3_N_4_. Reproduced with permission.^[^ ^202^ ^]^ Copyright 2023, Wiley‐VCH. k) Improvement of self‐polarization in PVDF for H_2_O_2_ evolution through CNTs incorporation. Reproduced with permission.^[^ ^205^ ^]^ Copyright 2023, Elsevier. l) Defect‐enhanced piezoelectric photocatalytic H_2_O_2_ generation in C_3_N_5_.^[^ ^206^ ^]^ Reproduced with permission. Copyright 2023, Elsevier.

Zhang et al. synthesized two MOFs, UiO‐66‐NH_2_ (Zr), and UiO‐66‐NH_2_ (Hf), whose piezo‐activity was significantly enhanced by increasing the strength of the electrostatic field.^[^ ^124^ ^]^ Applying mechanical force to the MOF material induces a piezoelectric field, which promotes charge separation and transfer rates, thereby enhancing catalytic activity. The performance of the Pt/Uio‐66‐NH_2_ (Hf) catalyst is greatly improved under ultrasound compared to the conditions without ultrasound or stirring. H_2_ production is optimal when the ultrasonic power gradually increased to 200 W and the ultrasonic frequency reaches 53 kHz. The catalysts demonstrated a significant H_2_ production capacity under the combined effects of ultrasound and light irradiation, achieving 1615 µmol g^−1^ h^−1^ for UiO‐66‐NH_2_ (Hf) and 740 µmol g^−1^ h^−1^ for UiO‐66‐NH_2_ (Zr), respectively (**Figure** **7**a). The calculated piezoelectric coefficient *d*_33_ showed that the *d*_33_ for Hf‐MOF (139 pm V^−1^) was significantly higher than that for Zr‐MOF (4 pm V^−1^). The higher *d*_33_ of Hf‐MOF is attributed to the greater polarity of Hf─O bonds compared to Zr─O bonds. This enhanced polarization contributes to the formation of ultrasound‐induced piezoelectric effects, which facilitate charge transport and mass transfer. As a result, UiO‐66‐NH_2_ (Hf) exhibits superior catalytic activity and cycling stability. Cao et al. synthesized a Zn, N co‐doped porous carbon piezocatalyst (Zn─N*_x_* ─C), obtained by pyrolysis of zeolitic imidazolium framework‐8 (ZIF‐8). Thus, Zn─N*_x_* ─C can be regarded as a derivative of MOFs.^[^ ^120^ ^]^ Zn─N*_x_* ─C exhibits superior piezocatalytic performance compared to ZIF‐8, achieving an H_2_ evolution rate of 6.29 mmol g^−1^ h^−1^ (Figure 7b). The *d*_33_ value for Zn─N*_x_* ─C is calculated to be 38 pm V^−1^. This high piezoelectric coefficient results from the asymmetric distribution of positive and negative charges around zinc atoms. The high specific surface area and numerous mesopores contribute to enhanced H_2_ evolution efficiency. The introduction of halogen atoms can alter the structure and electron distribution within MOFs. Zhao et al. prepared UiO‐66(Zr)‐F_4_ MOF, where F element reduces the bandgap and increases the asymmetry. This significantly enhances piezoelectricity, with a maximum H_2_ evolution rate reaching 35.7 µmol g^−1^ under 110 W ultrasound.^[^ ^156^ ^]^

Hu et al. employed thermal oxidation etching of bulk g‐C_3_N_4_ to synthesize ultra‐thin g‐C_3_N_4_ nanosheets (UT g‐C_3_N_4_).^[^ ^48^ ^]^ It has been reported that the increase in polar tri‐*s*‐triazine units along the planar axis of the layers enhances the dipole moment of g‐C_3_N_4_. When these tri‐*s*‐triazine units undergo stretching or compression due to strain, the positions of positive and negative charge centers shift, resulting in generation of in‐plane piezoelectric polarization (Figure 7c). The H_2_ evolution efficiency of UT g‐C_3_N_4_ is measured at 8.35 mmol g^−1^ h^−1^, which is 3.1 times greater than that of bulk g‐C_3_N_4_. This enhancement is attributed to the efficient harvesting and conversion of mechanical energy facilitated by its plate‐like structure. Moreover, under illumination, the piezo‐photocatalytic activity significantly improves, allowing H_2_ yield to reach 12.16 mmol g^−1^ h^−1^. Generally, electrons induced by piezoelectric effects can be transferred to the interface between the solution and solid, forming a depletion layer that drives electrochemical reactions. Under illumination, a more efficient exchange of electrons occurs between the piezoelectric surface and the electroactive species, leading to faster charge carrier transfer and enhanced electrochemical reactions due to the increased availability of piezoelectric charges during the photo‐piezoelectric catalytic process. Additionally, ultrasound cavitation can generate N and C defects that serve as active sites, further accelerating the adsorption and activation of H_2_O molecules. In piezoelectric catalysis, ultrasound is commonly utilized as an external source to deform materials. However, this approach can incur high energy costs since most piezoelectric catalysts are in powder form. PVDF, as a flexible film, plays a crucial role in absorbing weak mechanical energy, such as that from flowing water. The g‐C_3_N_4_/LiNbO_3_/PVDF membrane efficiently absorbs vibration energy from water flow, generating a piezoelectric field that facilitates electron transfer to the active sites, resulting in H_2_ production (Figure 7d).^[^ ^55^ ^]^

#### 4.1.3 CO_2_ Reduction

The excessive use of fossil fuels has led to a significant increase in global CO_2_ emissions, resulting in a dramatic rise in atmospheric CO_2_ levels and contributing to climate change and environmental degeneration. The conversion of CO_2_ into valuable chemicals presents an attractive yet challenging strategy to mitigate environmental pollution and address energy shortages. CO_2_ reduction has garnered considerable attention in recent years, particularly in the pursuit of achieving carbon neutrality. The transformation of CO_2_ into CO/CH_4_, as well as other high‐value products, involves multiple electron transfer pathways: converting CO_2_ to CO requires two electrons, while the conversion to CH_4_ necessitates eight electrons.^[^ ^195^ ^]^ Piezoelectricity offers an internal driving force that enhances electron transfer, positioning it as a promising technique to accelerate CO_2_ reduction.^[^ ^196^ ^]^ Additionally, piezoelectric carbon‐based materials, known for their excellent conductivity and mechanical strength, play a crucial role in advancing research in piezo‐catalytic CO_2_ reduction.

Utilizing the sheet‐like structure of g‐C_3_N_4_, stirring vibrations can be employed instead of ultrasound to induce deformation and activate the piezoelectric effect. Chen et al. synthesized ZnS‐loaded g‐C_3_N_4_ via a solid‐phase method, demonstrating outstanding piezo‐photoelectric catalytic activity for CO_2_ reduction, achieving a conversion of CO_2_ to CH_4_ with 95.7% selectivity. This remarkable performance is attributed to the strong piezoelectric field, the built‐in electric field at the interface, and enhanced light absorption capacity that facilitate the transformation of CO_2_ to CH_4_ during piezo‐photocatalysis (see Figure 7e).^[^ ^197^ ^]^ The combined effects of light and vibrations significantly promote charge carrier separation and transfer, thereby enhancing CO_2_ conversion. Zhu et al. prepared Au_25_ NCs/RCN, achieving a CO yield of 111.95 µmol g^−1^ h^−1^ under piezo‐photocatalysis. This production rate is 4.03 times and 11.74 times greater than that achieved through individual photocatalysis and piezo‐catalysis, respectively.^[^ ^196^ ^]^ Flexible PVDF films facilitate material cycling and efficiently absorb vibrational energy. Wang et al. incorporated CdS to increase the piezoelectric *β*‐phase content of PVDF through coordination between F^−^ and Cd^2+^ ions, resulting in the formation of an internal electric field at the heterojunction between CdS and PVDF (see Figure 7f).^[^ ^81^ ^]^ Furthermore, Ag nanoparticles deposited on CdS generate localized surface plasmon resonance (LSPR), which accelerates electron transfer under light irradiation. Consequently, the synthetic effects of piezoelectric potential, internal electric fields, and LSPR significantly enhance the reduction of CO_2_ to CO and CH_4_, yielding 240.4 and 15.3 µmol g^−1^ h^−1^, respectively. Notably, CH_4_ production occurs only when PVDF is present, which is attributed to the stronger driving force for electron transfer provided by the piezoelectric potential compared to the internal electric field and LSPR, enabling the transfer of at least eight electrons to react with CO_2_ to form CH_4_.

#### 4.1.4 H_2_O_2_ Production

In electrochemical processes, the production of H_2_O_2_ can occur via a one‐step, two‐electron transfer mechanism or a two‐step, one‐electron transfer mechanism, along with direct water oxidation reactions. The relevant equations are as follows:^[^ ^198^ ^]^

(6) $$O_{2}+2H^{+}+2e^{-}\to H_{2}O_{2}\left(+0.68\;V\;\mathrm{vs}\;\mathrm{NHE}\right)$$

(7)

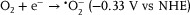



(8)

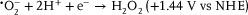

(9) $$2H_{2}O+2h^{+}\to H_{2}O_{2}+2H^{+}\left(+1.76\;V\;\mathrm{vs}\;\mathrm{NHE}\right)$$

The VB and CB of reported g‐C_3_N_4_ are 1.4 V versus NHE and −1.3 V versus NHE, respectively, indicating that the one‐step, two‐electron transfer mechanism is suitable for H_2_O_2_ generation.^[^ ^199^ ^]^ However, the piezoelectric O_2_ reduction reaction (ORR) occurs under ultrasonic conditions, where ultrasound generates localized heat and electrons that can rapidly reduce O_2_ to H_2_O_2_. Simultaneously, the ultrasound‐induced piezoelectric field facilitates energy band tilting to align with the ORR potential. Wang et al. reported that g‐C_3_N_4_ could produce 34 µmol h^−1^ of H_2_O_2_ under piezoelectric catalysis, where electron–hole pairs are separated in‐plane via ultrasonic‐derived piezoelectric polarization (Figure 7g).^[^ ^200^ ^]^ By designing the experiment under different atmospheres, it is proved that H_2_O_2_ is mainly originated from O_2_. The enhanced piezoelectric effect at an increased stirring speed further accelerates the generation of H_2_O_2_. The electrons are then transferred to the active sites, participating in both the two‐step one electron transfer and direct water oxidation reactions to form H_2_O_2_. The combined effects of piezoelectricity and photocatalysis enhance catalytic performance. The piezoelectric H_2_O_2_ evolution activity of g‐C_3_N_4_ with varying morphologies was investigated by Wang et al., who synthesized g‐C_3_N_4_ nanosheets, nanotubes and nanoparticles. Among these, g‐C_3_N_4_ nanotubes exhibited the highest H_2_O_2_ yield of 262 µmol g^−1^ h^−1^, which is 1.5 times and 6.2 times higher than that of nanosheets and hollow nanospheres, respectively. This enhanced performance is attributed to the ease of deformation of g‐C_3_N_4_ nanotubes under ultrasound, which induces a stronger piezoelectric potential that facilitates electron transfer (Figure 7h).^[^ ^54^ ^]^ While numerous reports highlight the enhancement of photocatalysis through the piezoelectric effect, it is essential to consider structural variations as well. Wang et al. explored the role of piezoelectricity in photocatalytic H_2_O_2_ production by designing g‐C_3_N_4_ modified with phosphorus (CN‐P), oxygen‐functionalized CN (CN‐OF), and cyano‐group (CN‐CA). The piezo‐photocatalytic H_2_O_2_ evolution yields improved compared to individual photocatalysis and piezocatalysis for CN, CN‐P, and CN‐OF. However, for CN‐CA, the piezo‐photocatalytic H_2_O_2_ production was six times lower than that achieved through photocatalysis alone. This discrepancy arises because the direction of piezoelectric polarization in CN‐CA opposes that in the other structures under ultrasound, inhibiting carrier separation and transfer in CN‐CA while promoting it in CN, CN‐P, and CN‐OF (Figure 7i).^[^ ^201^ ^]^ Li et al. reported on metal–organic cage‐coated gold nanoparticles anchored to graphitic carbon nitride (MOC‐AuNP/g‐C_3_N_4_), achieving a generation rate of 120.21 µmol g^−1^ h^−1^ for H_2_O_2_ via piezocatalysis. Theoretical calculations indicate that the metal–organic cage enhances the adsorption of O_2_ and H_2_O_2_. AuNP generates a strong polarization electric field that drives both holes and electrons. The H_2_O_2_ could be generated by 2e^−^ ORR and 4e^−^ WOR (see Figure 7j).^[^ ^202^ ^]^ Mg and Cl co‐doped g‐C_3_N_4_ exhibited a piezo‐photocatalytic yield of 1147.03 µmol h^−1^ for H_2_O_2_ production. The incorporation of Mg and Cl elements results in crystal distortion within g‐C_3_N_4_, enhancing its piezoelectric properties.^[^ ^203^ ^]^ The construction of an S‐scheme heterojunction between CdS and g‐C_3_N_4_ inhibits carrier recombination due to the built‐in electric field at the interface, achieving an impressive H_2_O_2_ yield rate of 23.44 mmol g^−1^ h^−1^.^[^ ^204^ ^]^ There are two pathways for H_2_O_2_ evolution: one involves h^+^ oxidizing H_2_O to form H_2_O_2_, while the other involves ^•^O_2_^−^ generated from the reaction between e^−^ and O_2_ undergoing subsequent chemical reactions to produce H_2_O_2_. Additionally, the flexibility of PVDF allows it to be employed for piezoelectric H_2_O_2_ generation. To enhance the *β*‐phase content, CNT/PVDF composites were constructed, achieving a *β*‐phase content improvement to 95%. Under ultrasonic irradiation, the composite yielded 1013 µmol of H_2_O_2_ (Figure 7k).^[^ ^205^ ^]^ Introducing defects is also a well‐established strategy to improve piezoelectric performance as they can narrow the bandgap and promote carrier transfer. Fu et al. prepared N‐defect C_3_N_5_ with CN groups, generating 1359 µmol g^−1^ h^−1^ of H_2_O_2_ under piezo‐photocatalysis (Figure 7l).^[^ ^206^ ^]^ KPFM and DFT calculations clarified that these defects enhance carrier transfer, as well as the adsorption and activation of O_2_. The combination of H_2_O_2_ and PMS activation could further achieve the efficient degradation of pollutants. A new type of piezoelectric covalent triazine based nanotube (CTN‐1) was synthesized by Jin et al. CTN‐1 facilitates the efficient synthesis of H_2_O_2_ from atmospheric water through the conversion of mechanical energy, achieving a remarkable piezocatalytic H_2_O_2_ evolution rate of 4115 µmol g⁻¹ h⁻¹. The external pressure decreases the band gap and conjugation of the present covalent triazine framework under high pressure is further increased, both of them contribute to strengthening charge migration.^[^ ^207^ ^]^

### 4.2 Piezoelectric Biomedical Treatments

The application of piezoelectric materials in anticancer therapy, nerve and bone regeneration and wound healing has been extensively documented, with carbon‐based materials playing a crucial role due to their low toxicity or non‐toxicity both in vitro and in vivo.^[^ ^29^, ^208^, ^209^ ^]^ Piezoelectric biomedical treatments involve the generation of ROS under external stimulation. These ROS can oxidize cellular components, leading to cell membrane disruption and subsequent cytoplasmic destruction. The piezoelectric‐induced Fenton‐like reaction is frequently employed in tumor treatment. Piezoelectrically generated holes can oxidize H_2_O into H_2_O_2_, which can then be activated to produce ^•^OH that effectively kills tumor cells. For carbon‐based piezoelectric materials, biocompatibility and toxicity are critical considerations, especially when used in applications such as implantable devices, tissue engineering, and drug delivery systems. Carbon‐based materials can exhibit cytotoxic effects depending on their surface chemistry and concentration. It has been reported that certain materials, such as glycine, may exhibit toxicity to cells, and the release of metal ions from MOFs can also exert toxic effects on cells,^[^ ^165^, ^167^ ^]^ However, these issues can be mitigated by surface functionalization. Modifying the surface of carbon‐based materials with biocompatible coatings or functional groups can reduce cytotoxicity and improve compatibility with biological tissues.

**Figure 8:**
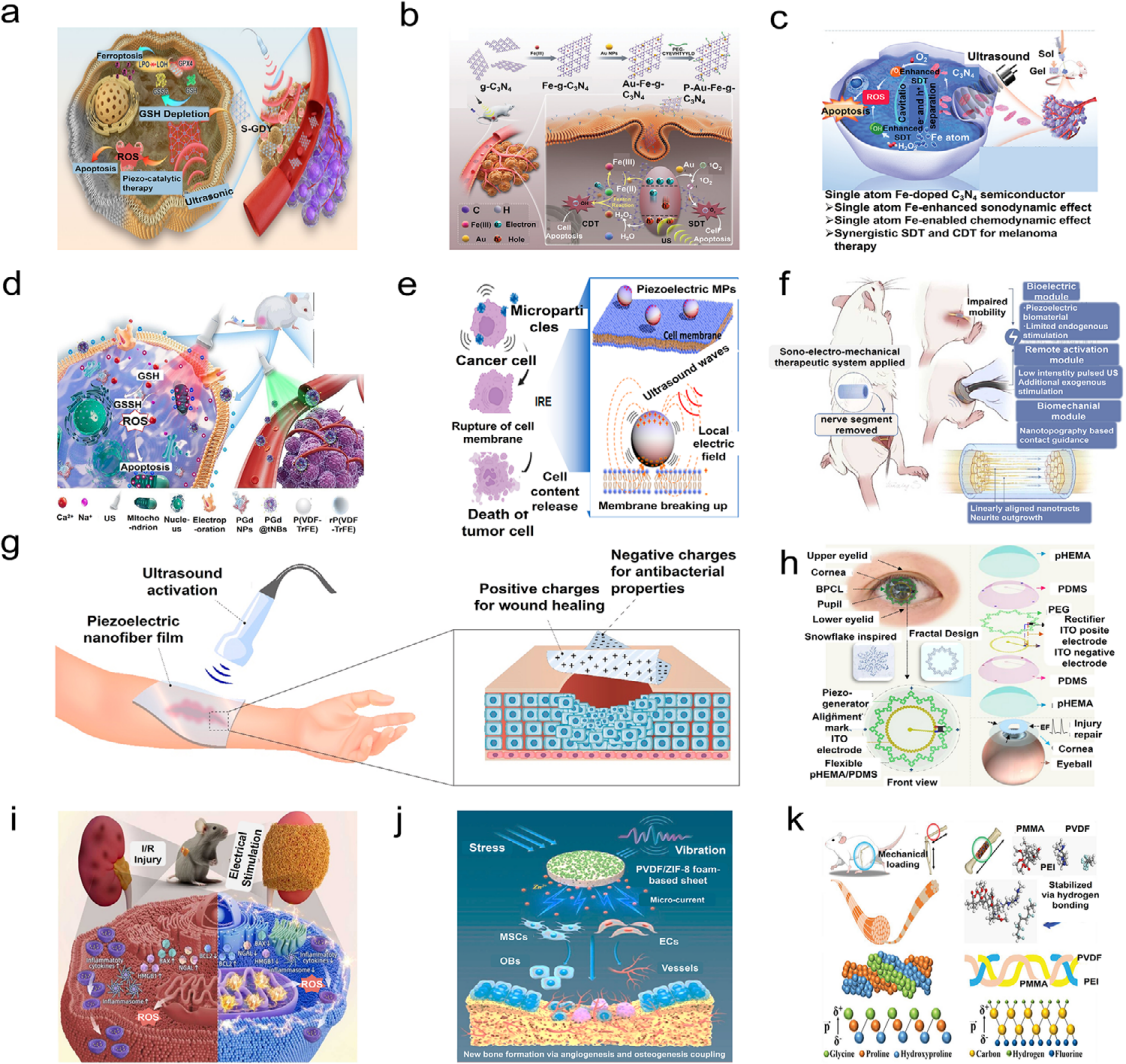
a) S‐GDY induces apoptosis in 4T1 cells via ROS. Reproduced with permission.^[^ ^210^ ^]^ Copyright 2023, The Authors, published by Springer Nature. b) P‐Au‐Fe‐g‐C_3_N_4_ enters the cell through the membrane and generates a Fenton‐like reaction under ultrasound, leading to apoptosis in HCT‐116 cells. Reproduced with permission.^[^ ^211^ ^]^ Copyright 2023, Wiley‐VCH. c) Combination of Fe‐g‐C_3_N_4_ with H_2_O_2_ generates ROS to kill B16F10 tumors. Reproduced with permission.^[^ ^212^ ^]^ Copyright 2023, Wiley‐VCH. d) PGd@tNBs induce ROS for apoptosis in SK‐BR3 cells via piezoelectric mechanisms. Reproduced with permission.^[^ ^213^ ^]^ Copyright 2023, The Authors, published by Springer Nature. e) P(VDF‐TrFE) generates an electric field that disrupts cell membranes. Reproduced with permission.^[^ ^214^ ^]^ Copyright 2023, American Chemical Society. f) Nerve regeneration facilitated by piezoelectric stimulation. Reproduced with permission.^[^ ^215^ ^]^ Copyright 2023, Elsevier. g) Piezoelectric wound repair and bacterial growth inhibition using PLLA films. Reproduced with permission.^[^ ^216^ ^]^ Copyright 2023, American Chemical Society. h) Snowflake‐inspired and blink‐driven flexible piezoelectric contact lenses for effective corneal injury repair. Reproduced with permission.^[^ ^217^ ^]^ Copyright 2023, The Authors, published by Springer Nature. i) PLLA/VB2 piezoelectric membranes for repairing renal I/R injury. Reproduced with permission.^[^ ^218^ ^]^ Copyright 2024, Elsevier. j) ZIF‐8 decorated PVDF facilitating piezoelectric bone regeneration. Reproduced with permission.^[^ ^219^ ^]^ Copyright 2023, Wiley‐VCH. k) PMMA/PEI/PVDF composites enhancing the expression of osteogenesis‐related genes for bone tissue regeneration. Reproduced with permission.^[^ ^220^ ^]^ Copyright 2023, Wiley‐VCH.

Wang et al. reported on sulfur‐doped graphdiyne (S‐GDY), which exhibits low toxicity to organisms. The ultrasound‐induced piezoelectric effect demonstrates exceptional nanozyme activity both in vitro and in vivo, potentially triggering apoptosis or ferroptosis in tumors (**Figure** **8**a).^[^ ^210^ ^]^ S‐GDY can catalyze H_2_O_2_ piezoelectrically to generate ^•^OH, which inactivates 4T1 tumor cells under ultrasonic conditions. Au‐deposited Fe‐doped g‐C_3_N_4_ (Au‐Fe‐g‐C_3_N_4_), with a size of ≈200 nm, can penetrate cell membranes when modified with CYEVHTYYLD and polyethylene glycol (PEG). Under ultrasound, electrons and holes are separated and transferred. Au particles served as active sites that accelerate electron transfer, reducing O_2_ to ^1^O_2_. Concurrently, holes oxidize H_2_O to H_2_O_2_, which is further decomposed into ^•^OH via the Fenton reaction involving Fe(II). The resulting Fe(III) is reduced back to Fe(II) by electrons. Consequently, both ^•^OH and ^1^O_2_ contribute to tissue necrosis and DNA damage, achieving an 85.6% cell apoptosis rate in HCT‐116 cells (Figure 8b).^[^ ^211^ ^]^ Similarly, single‐atom Fe‐doped g‐C_3_N_4_ (Fe‐C_3_N_4_) has been utilized for tumor treatment (Figure 8c).^[^ ^212^ ^]^ In vitro studies show that B16F10 mouse melanoma cells can uptake 97.29% of Fe‐C_3_N_4_, maintaining 90% cell viability, indicating its superior biocompatibility and biosafety. In the presence of Fe‐C_3_N_4_, H_2_O_2_, and ultrasound, the number of B16F10 cancer cells rapidly decreases due to the generated ROS acting as a potent driver of apoptosis. To enhance biocompatibility and biosafety, a gel composed of chitosan and 𝛽‐glycerophosphate (𝛽‐GP) was used to encapsulate Fe‐C_3_N_4_. When this composite was injected into B16F10 tumor‐bearing mice along with H_2_O_2_, the tumor inhibition rate reached as high as 98% under ultrasonic treatment. The piezoelectric effect has also been employed to treat breast cancer cells, specifically HER2‐positive SK‐BR3 cancer cells. Chen et al. synthesized PGd@tNBs nanoparticles composed of P(VDF‐TrFE) as piezoelectric materials, with DSPE‐PEG‐DOTA‐Gd enhancing hydrophilicity and DSPE‐PEG‐FITC providing fluorescent properties. Under ultrasound, the composite generates a substantial amount of ROS, particularly ^•^OH, thus achieving excellent antitumor efficacy both in vitro and in vivo while demonstrating biocompatibility to organisms (Figure 8d).^[^ ^213^ ^]^ In addition to ROS‐mediated mechanisms for eliminating cancer cells, the current generated by piezoelectric materials can directly induce cell death. P(VDF‐TrFE) microparticles can induce an irreversible electroporation effect on 4T1 breast cancer cells under ultrasound exposure, leading to cell rupture and death. This phenomenon occurs because P(VDF‐TrFE) generates a localized electric field that disrupts the cell membrane and homeostasis due to its low surface potential (Figure 8e).^[^ ^214^ ^]^

Piezoelectric tissues repair technologies have emerged as alternatives to traditional surgical methods that are often time‐consuming. Piezoelectric materials generate ultrasound‐activated electric stimulation that promotes cell regeneration. Pi et al. utilized piezoelectric nanotracks made from PCL and PVDF to treat a rat model with a 15 mm sciatic nerve defect. The rats restored complex motor functions and achieved axonal maturity comparable to standard autograft treatments (Figure 8f).^[^ ^215^ ^]^ Furthermore, piezoelectric materials have been applied for wound‐healing. PLLA piezoelectric nanofibers were placed into skin defects on the backs of mice under ultrasound, leading to skin regeneration after 14 days. The PLLA film deforms under external forces, hence, generating polarization charges on opposite sides. Negative surface charges suppress bacterial growth, while positive surface charges promote skin regeneration (Figure 8g).^[^ ^216^ ^]^ Piezoelectricity has also shown promise in repairing corneal injuries. Yao et al. developed a flexible blink‐driven piezoelectric contact lens (BPCL) made from Al/electret/Al film and ITO/PET film. By using mouse and rabbit models with moderate corneal injuries, it was found that BPCL significantly improved corneal healing. The underlying mechanism involves pressure generated between the eyelid and cornea during blinking, driving the BPCL and producing a pulse voltage signal that stimulates effective repair through orderly epithelial alignment while alleviating stromal fibrosis (Figure 8h).^[^ ^217^ ^]^ Feng et al. synthesized PLLA combined with VB_2_‐enhanced piezoelectric composite nanofibrous membranes for repairing renal ischemia/reperfusion (I/R) injury. This nontoxic and biocompatible film was wrapped around the injured kidney before implantation into mice, resulting in significant improvements in kidney function. The study suggests that alleviation of renal tubule injury, promotion of cell regeneration, and relief from interstitial fibrosis may be achieved by preserving mitochondrial structure and function and while rescuing superoxide dismutase through the piezoelectric effect (Figure 8i).^[^ ^218^ ^]^ Bone defects can also be regenerated using piezoelectricity. ZIF‐8 decorated PVDF has been reported to facilitate bone regeneration under ultrasound exposure. PVDF generates a piezoelectric potential that influences the trajectories of released Zn^2+^ ions from ZIF‐8, enriching Zn^2+^ around the PVDF surface while inducing microcurrent stimulation that guides vascularized bone regeneration. Hence, new bone formation occurs via angiogenesis and osteogenesis coupling (Figure 8j).^[^ ^219^ ^]^ PMMA/PEI/PVDF was combined by H bonding. The composites were developed to mimic the viscoelastic and piezoelectric microenvironment of bone tissue. When implanted into bone defects of similar size, this composite significantly enhanced the expression of osteogenesis‐related genes in human bone marrow stem cells in vitro while facilitating cortical and spongy bone regeneration though activation of Piezo1 protein linked to the mechanotransduction signaling pathway (Figure 8k).^[^ ^220^ ^]^ T. Chorsi et al. fabricated piezoelectric nanofibers embedded with glycine crystals within PCL, creating a biodegradable ultrasound transducer designed for delivering chemotherapeutic drugs directly to the brain, effectively doubling survival time in mice with orthotopic glioblastoma models.^[^ ^221^ ^]^

### 4.3 Nanogenerators and Sensors

**Figure 9:**
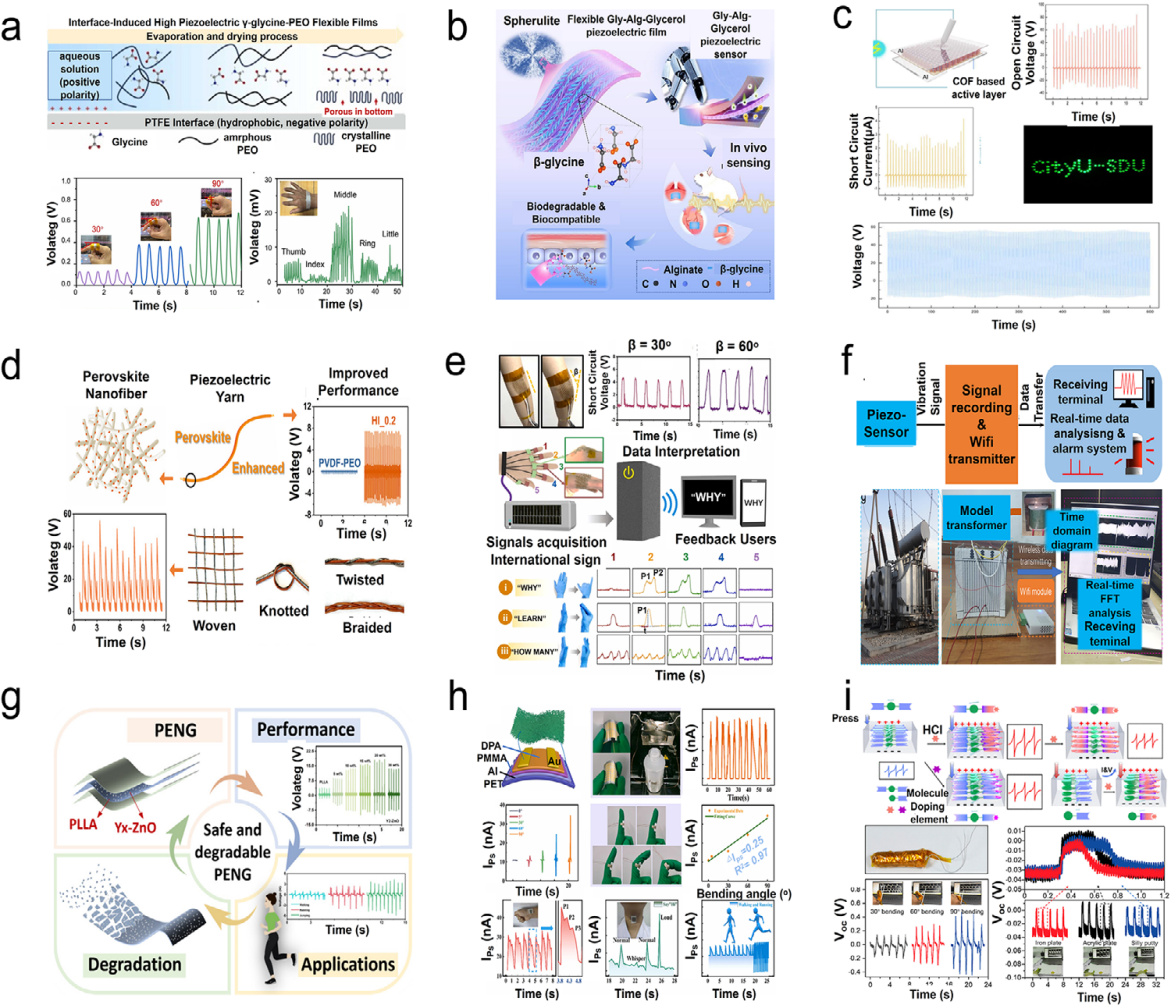
a) Schematic illustration of the evaporation and drying process of glycine‐PEO aqueous solutions on PTFE interfaces and the resulting piezoelectric voltage generation. Reproduced with permission.^[^ ^226^ ^]^ Copyright 2024, Elsevier. b) Piezoelectric voltage output of Gly‐Alg‐Glycerol film in different body positions. Reproduced with permission.^[^ ^227^ ^]^ Copyright 2024, The Authors, published by Oxford University Press. c) Al/COF/Al nanogenerator generating current and voltage to power LEDs. Reproduced with permission.^[^ ^132^ ^]^ Copyright 2024, The Authors, published by Wiley‐VCH. d) Enhanced piezoelectric signals from perovskite‐decorated PVDF yarns. Reproduced with permission.^[^ ^228^ ^]^ Copyright 2024, The Authors, published by American Chemical Society. e) Voltage generated by elbow movements at different angles and the signals utilized in a smart gesture recognition system. Reproduced with permission.^[^ ^229^ ^]^ Copyright 2024, Elsevier. f) Demonstration of transformer vibration monitoring applications based on a piezoelectric fiber sensor made from PC@PVDF‐TrFE. Reproduced with permission.^[^ ^231^ ^]^ Copyright 2023, American Chemical Society. g) Piezoelectric and degradable performance of ZnO/PLLA films for applications in body motion detection. Reproduced with permission.^[^ ^232^ ^]^ Copyright 2024, American Chemical Society. h) Piezoelectric testing of flexible PVDF‐OFET‐PZTs in various body positions. Reproduced with permission.^[^ ^233^ ^]^ Copyright 2023, Elsevier. i) Charge distribution, changes in piezoelectric performance after doping, and voltage generation of the wearable finger‐shaped sensor at different bending angles and when touching various hard materials. Reproduced with permission.^[^ ^237^ ^]^ Copyright 2023, American Chemical Society.

Since Wang et al. first reported the ZnO piezoelectric nanogenerator in 2010, numerous researchers have entered this field of study.^[^ ^222^, ^223^ ^]^ Piezoelectric materials deform under compression, and the induced piezoelectric potential drives electron transfer to produce current. Many carbon‐based piezoelectric materials, particularly biodegradable polymers, exhibit greater flexibility than inorganic materials, allowing them to deform and generate electrical signals under low forces.^[^ ^224^ ^]^ Recently, these types of piezoelectric polymers have been applied in pressure nanogenerators and sensors. It has been reported that composite membranes of chitosan and 𝛽‐glycine can be obtained after stripping from a Petri dish.^[^ ^225^ ^]^ In the subsequent step, electron beam evaporation and shadow mask methods were employed to deposit the gold electrode patterns. Wires are then attached to both sides of the deposited electrode, and the sensor is encapsulated using Kapton tape. The results indicate that the sensor can produce an output voltage of 190 mV at the pressure of 60 kPa. Yu et al. prepared a glycine‐PEO composite film with a *d*_33_ of ≈8.2 pC N^−1^. This film can generate a maximum voltage of 5 V and can be utilized as a wearable sensor for monitoring joint movement. When attached to the back of a finger or hand, it can detect and recognize movement signals for various bending angles or different fingers, demonstrating its potential for gesture recognition applications. The glycine‐PEO composite film exhibits excellent mechanical‐to‐electrical energy conversion and sensing performance in wearable and implantable applications (**Figure** **9**a).^[^ ^226^ ^]^ A novel biocompatible and biodegradable piezoelectric film composed of *β*‐glycine, alginate, and glycerol (Gly‐Alg‐Glycerol) was developed, showcasing exceptional sensing performance both in vitro and in vivo. Notably, this film features a single, monolithic *β*‐glycine spherulite within the alginate matrix, contrasting with the commonly observed multiple spherulites. This unique structure endows the film with superior piezoelectric properties, including a high piezoelectric constant of 7.2 pC N^−1^ and a sensitivity of 1.97 mV kPa^−1^. The film demonstrates remarkable sensitivity and stability, consistently generating stable output voltages in response to various pressures and bending deformations, reaching a maximum of 0.95 V under 80 N. In vitro studies indicate that Gly‐Alg‐Glycerol promotes the proliferation of mesenchymal stem cells. Additionally, the piezoelectric sensor accurately detects subtle pulse signals from the human carotid artery, sound wave signals and shear stress from different directions (Figure 9b).^[^ ^227^ ^]^ Gu et al. prepared an Al/COF/Al nanogenerator that generated a maximum voltage of ≈80 V and a short‐circuit current of 2.4 µA. The COF‐based PENG was applied to directly power an array of LEDs, demonstrating superior long‐term durability of Al/COF/Al nanogenerator without any noticeable decrease in piezoelectric output voltage (Figure 9c).^[^ ^132^ ^]^ Although organic films used as cores in nanogenerators exhibit excellent piezoelectric voltage performance, durability is also crucial for achieving industrial applications. To enhance stability, Wu et al. fabricated a single yarn of CsPbI_2_Br‐decorated PVDF nanofibers with excellent mechanical properties. The nanofibers generated an output voltage of 8.3 V and a current of 1.91 µA under pressure, which can be used to charge capacitors for powering electronics. The PENG yarn incorporating HI 0.45 nanofibers achieved the best output voltage of 8.4 V, an output current of 1.92 µA, and a power density of 281 µW cm^−^^3^ (Figure 9d).^[^ ^228^ ^]^ Self‐poled PENG devices were successfully synthesized by incorporating oriented ionic salt‐montmorillonite (IS‐MMT) co‐fillers into a PVDF matrix (MPENG‐180), followed by 3D printing. When MPENG‐180 was used as a flexible electronic skin for intelligent sensing mounted on joints, these 3D‐printed PENGs accurately detected muscle movements. The MPENG‐180 model exhibited high sensitivity to bending angles, with output voltages increasing from ≈4 to 6 V as the elbow bending angle increased. This highlights the potential of these self‐powered sensors to capture biomechanical energy and monitor human physiological motion. Moreover, MPENG‐120 and MPENG‐180 were worn on the metacarpophalangeal (MCP) and interphalangeal (PIP) joints. The generated voltage signal amplitudes and shapes were attributed to different mechanical deformations and strain rates experienced by sensors worn on distinct finger joints. Additionally, by analyzing output values and peak patterns of electrical signals, various joint exercise intentions and angles could also be distinguished (Figure 9e).^[^ ^229^ ^]^ The phenomenon is similar to that observed with PLLA combined with CO_2_‐based polyurea (PU) film.^[^ ^230^ ^]^ The piezoelectric sensor was securely attached to various body parts including the neck, wrist, elbow, knee, and the bottom of the insole. It effectively monitored arterial pulsation, swallowing, joint bending, and walking activities. The varying motion behaviors exerted different forces on the sensor, thereby influencing the voltage output.

The PC@PVDF‐TrFE core‐shell nanofiber could generate an output voltage of 126 V, sufficient to easily illuminate 50 white LEDs through mechanical bending due to its high energy‐harvesting performance. PC@PVDF‐TrFE can also be integrated into a wireless transformer vibration monitoring system to enable real‐time monitoring and alarm functions for power equipment. When tested on real transformers, the sensor generated a piezoelectric response of ≈5 V after activation by an electromagnet, successfully defecting vibration signals from operational power transformers (Figure 9f).^[^ ^231^ ^]^

Biodegradable PLLA film embedding yttrium (Y)‐doped ZnO film (Y‐Z‐PLLA) generated an open‐circuit voltage of 17.52 V, a short‐circuit current of 2.45 µA, and an instantaneous power density of 1.76 µW cm^−2^ under an external compressive force. Y‐Z‐PLLA could be completely degraded in NaOH solution. To simulate practical application environments, the packaged PENGs were affixed to the soles of feet during activities such as walking, running, and jumping with the observed signals varying according to different motions (Figure 9g).^[^ ^232^ ^]^ A PVDF membrane combined with an organic field‐effect transistor (OFET) (PVDF‐OFET‐PZTS) was constructed. Additionally, this sensor was attached to a finger to detect signals generated by bending at different angles in real‐time. At each bending angle, the electrical signal exhibited a clear linear relationship. Flexible PVDF‐OFET‐PZTS devices were mounted on various body parts, including the wrist, throat, and sole of the foot, to collect pulse signals, vocal cord vibration signals, and walking signals. These devices effectively captured diverse physiological signals when mounted on different areas of the body (Figure 9h).^[^ ^233^ ^]^ Tajitsu et al. prepared a high‐efficiency medical catheter based on PLLA electrospun nanofibers with enhanced piezoelectric properties.^[^ ^234^ ^]^ Subsequently, two electrodes with diameters of 40 µm were coated on the top surface of the fiber to act as actuators. PLLA fibers were fabricated using electrospinning technology followed by post‐treatment strain was observed when an AC voltage ranging from −300 to 300 V was applied. PLLA nanofibers were fabricated using electrospinning technology followed by post‐treatment involving slow annealing and cooling processes to increase the crystallinity from 70% to 88%. Nanofibers were sandwiched between molybdenum electrodes on either side to create biodegradable force sensors. For encapsulation purpose, a PLLA layer force transducer was implanted intraperitoneally for monitoring intraperitoneal pressure in mice.^[^ ^235^ ^]^ The pressure response in the mouse's abdomen could be observed as it periodically compressed and relaxed.

Phenylalanine‐phenylalanine (FF) nanotubes demonstrated good piezoelectric properties with a piezoelectric coefficient *d*_15_ of 46.6 pm V^−1^. When subjected to a force of 42 N, they generated an output voltage of 2.8 V, a short‐circuit current of 37.4 nA, along with power generation capability reaching 8.2 nW, sufficient for operating multiple liquid crystal display panels.^[^ ^236^ ^]^ Acid doping (HCl, HBr, or HI) was applied to PLLA films. Among these variants, HCl‐doped PLLA (molar ratio 1:1) exhibited the largest enhancement in piezoelectricity due to the increased charge asymmetry induced by ─NH*─ groups accepting protons from HCl, thus enabling piezoelectric energy harvesters to produce voltages up to 3.4 V with currents reaching 80 nA, surpassing other doped PLLA variants. However, increasing HCl concentration could cause charge counteraction, negatively affecting the piezoelectricity. Therefore, further investigations into piezoelectric energy harvesters as flexible electromechanical coupling devices were conducted, specifically focusing on finger‐shaped sensors capable of detecting contact signals in all directions, where bending from 30° to 90° correspondingly increased generated voltage from 0.2 to 0.4 V. Additionally, when this wearable sensor contacted various hard materials, distinct piezoelectric voltage signals were recorded (Figure 9i).^[^ ^237^ ^]^

## 5 Challenges and Prospects

The development of emerging technologies and materials aims to benefit humanity. Carbon‐based piezoelectric materials have been extensively investigated across various fields. However, they have yet to achieve widespread practical applications. Below, we outline some key limitations and propose potential future directions for research and development.

1. Insufficient efficiency: Carbon‐based piezoelectric materials with high specific surface areas possess numerous pores that enhance contact and reaction with molecules. However, these same properties can inhibit their secondary utilization, as molecules can occupy the most active sites, particularly in environmental remediation applications. Pollutants or by‐product molecules may be tightly adsorbed onto active sites due to electrostatic interactions, resulting in generally low catalytic efficiency during secondary treatment compared to initial treatment. To address this issue, post‐treatment processes such as heating are required, which increases associated costs. Additionally, the presence of deep‐level defects can lead to the recombination of electron and hole transfer, significantly impacting the catalytic performance. Although strategies such as metal loading, heterojunction formation, and heteroatom doping exist to improve efficiency, their effects remain limited. Preparing carbon‐based piezoelectric materials with precisely controlled atomic‐level structures is a promising approach that could enhance charge carrier separation due to reduced transfer distances.
2. Limited external force sources: Current research predominantly utilizes ultrasound as an external force to induce catalytic activity based on cavitation effects, applicable in contaminant removal, water splitting, CO_2_ reduction, and biomedical treatments. However, the efficacy of ultrasonic effects is confined to relatively small areas, posing challenges for broader applications. Ball milling provides a more scalable source of mechanical force for driving piezoelectric catalysis and has been successfully applied in pollutant decomposition and chemical synthesis using inorganic piezoelectric materials. Therefore, ball milling‐induced piezoelectric catalysis represents a potential avenue for expanding the applications of carbon‐based piezoelectric materials.
3. Weak piezoelectric properties: A comparison between inorganic and carbon‐based piezoelectric materials is detailed in **Table** **1**. Inorganic piezoelectric materials, such as BaTiO_3_ (90 pC N–1), PbTiO3 (79.1 pC N–1), BiFeO_3_ (100 pm V–1), and ZnO (33.3 pm V–1), generally exhibit higher piezoelectric coefficients compared to their carbon‐based counterparts.^[^ ^238^ ^]^ The reported piezoelectric coefficients of many carbon‐based materials are relatively low, which can result in reduced charge carrier separation and transfer rates. However, recent advancements in material engineering and functionalization have shown potential for enhancing these coefficients. For example, morphologically engineered g‐C3N4 has demonstrated d33 values approaching 524 pm V–1, indicating significant progress in bridging the performance gap between carbon‐based and inorganic piezoelectric materials. The irregular orientation of many piezoelectric domains further hinders charge carrier separation. By applying an external polarizing voltage to piezoelectric materials, the orientation of these domains can be aligned, thereby enhancing charge carrier separation and transfer efficiency. Consequently, employing an internal polarized electric field is a promising approach due to the structural stabilization it offers for carbon‐based materials.
4. Mechanical stability under prolonged stress: Carbon‐based materials, such as graphene and CNTs, are renowned for their exceptional mechanical properties, including high tensile strength and flexibility. However, their performance under prolonged mechanical stress, especially in dynamic applications involving cyclic loading, remains a concern. Over time, repeated mechanical loading can induce fatigue, leading to microstructural changes such as crack initiation and propagation.^[^ ^239^ ^]^ These changes can degrade the piezoelectric properties, reducing the material's effectiveness in applications such as sensors and actuators. To address this limitation, researchers are exploring composite materials that incorporate carbon‐based elements with polymers or ceramics to enhance durability. These composites can distribute stress more evenly, reducing the likelihood of fatigue. Additionally, surface treatments and functionalization strategies can improve the interfacial bonding between carbon materials and their matrices, further enhancing mechanical stability.^[^ ^240^ ^]^ A comprehensive understanding of the underlying mechanisms of fatigue and the development of targeted strategies to mitigate its effects are crucial for ensuring the long‐term reliability of carbon‐based piezoelectric devices.
5. Scaling up production: Scaling up the production of carbon‐based materials from laboratory‐scale syntheses to industrial levels presents significant challenges. Techniques such as CVD and liquid‐phase exfoliation, while effective at small scales, often struggle to maintain the uniformity and quality required for large‐scale applications.^[^ ^241^ ^]^ Variations in thickness, purity, and defect density can adversely affect the piezoelectric properties, leading to inconsistent performance. To overcome these challenges, researchers are actively developing modified synthesis processes that enhance scalability without compromising material quality. Additionally, continuous production methods, such as roll‐to‐roll processing, are being explored to increase throughput and reduce production cost.^[^ ^242^ ^]^ Ensuring consistent material properties during scale‐up is critical for the commercialization of carbon‐based piezoelectric materials, as it directly impacts their reliability and performance in practical applications.
6. Control over piezoelectric coefficients: Achieving precise control over the piezoelectric coefficients of carbon‐based materials is a complex challenge due to their inherent sensitivity to subtle structural characteristics. Factors such as defect density, layer stacking, and chemical functionalization can significantly influence the piezoelectric response.^[^ ^243^ ^]^ Variability introduced during synthesis and processing can lead to inconsistencies in performance, hindering the development of reliable devices. Advanced characterization techniques, such as Raman spectroscopy, atomic force microscopy, and electron microscopy, are essential for elucidating critical structure–property relationships. These techniques provide insights into how specific structural features affect piezoelectric behavior, thereby guiding the optimization of synthesis processes. Additionally, computational modeling and machine learning approaches are being increasingly employed to predict material properties and design materials with tailored piezoelectric responses.^[^ ^244^ ^]^ By gaining a deeper understanding of the factors influencing piezoelectric coefficients, researchers can develop strategies to achieve consistent and predictable performance in carbon‐based piezoelectric materials.

**Table 1:** Comparison of piezoelectric coefficients for selected inorganic and carbon‐based materials.

Piezoelectric material	Piezoelectric coefficient [pm V^−1^]	Refs.
Ultrathin g‐C_3_N_4_ nanosheets	524	[48]
g‐C_3_N_4_ hollow nanotubes	34.99	[54]
N‐doped GDY	14.84	[140]
PVDF	23	[78]
HOCH_2_(CF_2_)_3_CH_2_OH	138	[113]
ZIF‐90	46	[127]
COF City‐U14	20.9	[132]
Graphene oxide	0.24	[150]
Graphene sheet	0.124	[145]
CNT	0.107	[154]
Hf‐MOF	139	[124]
Zn─N*_x_* ─C	38	[120]
FF nanotubes	46.6	[236]
KNbO_3_	29.3	[238]
ZnO	33.3	[238]
BiFeO_3_	100	[238]
BaTiO_3_	90	[238]

## 6 Conclusions

This review provides a comprehensive survey of the piezoelectric potential of carbon‐based materials across various applications. It highlights recent advancements in piezocatalytic activities, nanogenerators, sensors, and biomedical treatments driven by piezoelectric effects. The analysis encompasses fundamental mechanisms, such as mechanical force‐induced polarization, and offers a detailed overview of carbon‐based piezoelectric materials, including their distinctive structural characteristics. A key focus is placed on elucidating the influence of piezoelectric potential on charge carrier generation and separation in carbonyl piezocatalysts. This is contextualized within applications such as environmental remediation, hydrogen evolution, CO_2_ reduction, and the development of innovative biomedical devices. Finally, the review critically assesses current limitations and proposes potential solutions aimed at enhancing the practical applicability of carbon‐based piezoelectric materials, while highlighting promising avenues for future research.

## Conflict of Interest

The authors declare no conflict of interest.
